# Recombinant mycobacterial DNA-binding protein 1 with post-translational modifications boosts IFN-gamma production from BCG-vaccinated individuals’ blood cells in combination with CpG-DNA

**DOI:** 10.1038/s41598-024-58836-8

**Published:** 2024-04-21

**Authors:** Yuriko Ozeki, Akira Yokoyama, Akihito Nishiyama, Yutaka Yoshida, Yukiko Ohara, Tsukasa Mashima, Chikako Tomiyama, Amina K. Shaban, Atsuki Takeishi, Mayuko Osada-Oka, Takehiro Yamaguchi, Yoshitaka Tateishi, Jun-ichi Maeyama, Mariko Hakamata, Hiroshi Moro, Toshiaki Kikuchi, Daisuke Hayashi, Fumiko Suzuki, Toshiko Yamamoto, Sumiko Iho, Masato Katahira, Saburo Yamamoto, Sohkichi Matsumoto

**Affiliations:** 1 Department of Bacteriology, Niigata University School of Medicine, 1-757, Asahimachi-Dori, Chuo-Ku, Niigata, 951-8510 Japan; 2 Department of Respiratory Medicine, Graduate School of Medicine, University of Tokyo, 7-3-1, Hongo, Bunkyo-Ku, Tokyo, 113-8654 Japan; 3 Institute of Advanced Energy, Kyoto University, Gokasho, Uji, Kyoto, 611-0011 Japan; 4 Graduate School of Health Sciences, Niigata University, 2-746, Asahimachi-Dori, Chuo-Ku, Niigata, 951-8518 Japan; 5 Food Hygiene and Environmental Health, Division of Applied Life Science, Graduate School of Life and Environmental Sciences, Kyoto Prefectural University, 1-5, Shimogamo-Nakaragi-Cho, Sakyo-Ku, Kyoto, 606-8522 Japan; 6 Department of Bacteriology 1, National Institute of Infectious Disease, 1-23-1, Sinjuku-Ku, Tokyo, 162-8640 Japan; 7 Reseach Center for Biological Products in the Next Generation, National Institute of Infectious Diseases, 4-7-1, Musashimurayama, Tokyo, 208-0011 Japan; 8 Department of Respiratory Medicine and Infectious Disease, Niigata University Graduate School of Medical and Dental Sciences, 1-757, Asahimachi-Dori, Chuo-Ku, Niigata, 951-8510 Japan; 9 Central Laboratory, Japan BCG Laboratory, 3-1-5 Matsuyama, Kiyose, Tokyo, 204-0022 Japan; 10 Faculty of Medical Sciences, University of Fukui, 23-3, Matsuokashimoaizuki, Eiheiji-Cho, Yoshida-Gun, Fukui, 910-1193 Japan; 11 Louis Pasteur Center for Medical Research, 103-5 Tanaka Monzen-cho, Sakyo-ku, Kyoto, 606-8225 Japan; 12 Laboratory of Tuberculosis, Institute of Tropical Disease, Universitas Airlangga, Kampus C JI. Mulyorejo, Surabaya, 60113 Indonesia; 13 Division of Research Aids, Hokkaido University Institute for Vaccine Research and Development, Kita 20, Nishi 10, Kita-Ku, Sapporo, 001-0020 Japan; 14 Department of Mycobacteriology, Leprosy Research Center, National Institute of Infectious Diseases, 4-2-1 Aobacho, Higashi-Murayama, Tokyo, 189-0002 Japan

**Keywords:** Biotechnology, Immunology, Microbiology

## Abstract

Tuberculosis remains a large health threat, despite the availability of the tuberculosis vaccine, BCG. As BCG efficacy gradually decreases from adolescence, BCG-Prime and antigen-booster may be an efficient strategy to confer vaccine efficacy. Mycobacterial DNA-binding protein 1 (MDP1, namely Rv2986c, hupB or HU) is a major *Mycobacterium tuberculosis* protein that induces vaccine-efficacy by co-administration with CpG DNA. To produce MDP1 for booster-vaccine use, we have created recombinant MDP1 produced in both *Escherichia coli* (eMDP1) and *Mycolicibacterium smegmatis* (mMDP1), an avirulent rapid-growing mycobacteria. We tested their immunogenicity by checking interferon (IFN)-gamma production by stimulated peripheral blood cells derived from BCG-vaccinated individuals. Similar to native *M. tuberculosis* MDP1, we observed that most lysin resides in the C-terminal half of mMDP1 are highly methylated. In contrast, eMDP1 had less post-translational modifications and IFN-gamma stimulation. mMDP1 stimulated the highest amount of IFN-gamma production among the examined native *M. tuberculosis* proteins including immunodominant MPT32 and Antigen 85 complex. MDP1-mediated IFN-gamma production was more strongly enhanced when combined with a new type of CpG DNA G9.1 than any other tested CpG DNAs. Taken together, these results suggest that the combination of mMDP1 and G9.1 possess high potential use for human booster vaccine against tuberculosis.

## Introduction

Tuberculosis (TB) remains a serious threat to human health. World Health Organization (WHO) reported that in 2021, 10.6 million people developed TB and 1.6 million people died from the disease^1^. Established in the early twentieth century at the Pasteur Institute^2^, *Mycobacterium bovis* Bacille Calmette-Guérin (BCG) is currently the only approved vaccine against tuberculosis. It is primarily given to infants in accordance with the WHO recommendation^3^, and is recorded as one of the most widely administered vaccine to date.

BCG induces long term protective efficacy of 5–15 years whereby it prevents disseminated tuberculosis in children. However, the efficacy gradually wanes over time following vaccination^4^. This has necessitated the current urgency to develop more efficient and long-lasting vaccines^5^. However, the effects of newly developed vaccines have not been able to surpass BCG efficacy. For instance, the most anticipated vaccine that has advanced to Phase 2b of clinical trials was reported to have an effect of only 54%^6^. BCG is a live attenuated vaccine that shares many immunodominant antigens with its parent *M. tuberculosis*, except for a few antigens, such as ESAT6 and CFP10. In general, live vaccines tend to be more effective than inactivated vaccines that contain component vaccines. Therefore, developing inactivated vaccines that can exceed the efficacy of live BCG presents a significant challenge.

Aside from tuberculosis and leprosy, BCG is known to have broad protective effects against other non-related infectious diseases and cancers. Epidemiological studies showed that BCG vaccination reduces child mortality from causes other than tuberculosis in Sweden and African countries^7,8^, as well as the prevalence of respiratory diseases caused by viral infections in elderly individuals in Greece^9^. The immunological memory of innate immunity, often referred to as “trained immunity” or the shift to Th1-type immunity following vaccination, may explain the beneficial broad effects of BCG against other challenging infectious diseases^10^. In fact, during the recent coronavirus disease 2019 (COVID-19) pandemic, it was observed that BCG vaccination is inversely associated with the prevalence and mortality from COVID-19^9^.

Mycobacterial DNA-binding Protein 1 (MDP1, namely Rv2986c, hupB, or HU) is a major protein in *M. tuberculosis* located both inside and on the surface of the bacilli and is considered a candidate vaccine antigen^11^. MDP1 has a unique intrinsically disordered C-terminal region^12,13^, where post-translational modification occurs on the rich lysin (Lys) residues^14^. Similarly, Heparin-binding hemagglutinin (HBHA), a secreted protein in *M. tuberculosis*, is highly methylated^15^. Importantly vaccination with HBHA only induces protection when the protein was purified from *M. tuberculosis* or BCG but not recombinant from *Escherichia coli* (*E. coli*)^16^. This is because the later lacks mycobacteria-specific post-translational modification^15^. Interestingly HBHA and MDP1 share enzymes which methylate their lysins resides^17^. However, the immunological effect of post-translational modification on MDP1 has not been elucidated so far.

Adjuvants are definitively critical for induction of vaccine efficacy^18,19^. Interestingly, the antigenicity of MDP1- a DNA-binding protein- is augmented when combined with CpG-DNA, which activates TLR9 signaling pathway^20,21^. We previously showed that co-administration of MDP1 and *M. tuberculosis* genomic DNA conferred protection against *M. tuberculosis* infection in mice^11^. For vaccine development, synthetic counterparts of bacterial CpG-DNA should be considered as potential alternatives to using *M. tuberculosis* DNA. In addition to the three well-known types of CpG-DNA designated class A, B and C, we reported the synthesis of a novel CpG- DNA designated G9.1, which can be utilized as MDP1 adjuvant.^22,23^. The combination of *E. coli*-produced recombinant MDP1 (eMDP1) and G9.1 demonstrated significant protection against *M. tuberculosi*s challenge in BCG-primed guinea pig^22^. However, it remains uncertain whether G9.1 is a superior adjuvant for MDP1 compared to other immune-stimulatory CpG-DNAs.

For human vaccine use, it is difficult to prepare native proteins from *M. tuberculosis*, which requires handling under biosafety level 3 pathogen. In this study, we employed biosafety level 1 bacteria as hosts to create recombinant MDP1 that is suitable for vaccine development. We successfully expressed MDP1 in both *E. coli* (eMDP1) and *Mycolicibacterium smegmatis,* which was renamed from *Mycobacterium smegmatis*, an avirulent rapid grower among mycobacteria (mMDP1). Our efforts involved optimizing expression efficiency and characterizing the physical properties of eMDP1 and mMDP1. Subsequently, we evaluated the immunological properties of eMDP1 and mMDP1, both with and without various types of CpG-DNAs. Interferon (IFN)-gamma production plays a crucial role in host protection mediated by vaccine against tuberculosis in both animal and humans. It serves as a key marker of vaccine efficacy against tuberculosis^24–26^. Therefore, we measured IFN-gamma production using whole peripheral blood or peripheral blood mononuclear cells (PBMC) derived from individuals previously vaccinated with BCG and eligible for booster vaccination. Our comprehensive analysis indicated that combination of mMDP1 and G9.1 has high potential for human vaccine use against tuberculosis.

## Results

### Creating monoclonal antibodies (mAb) recognizing MDP1

To evaluate the nature of recombinant MDP1 as a tuberculosis vaccine candidate, we obtained two types of monoclonal antibodies (mAbs). The first mAb recognizes amino acid sequence of MDP1, while the second mAb recognizes post-translationally modified MDP1.

We immunized BALB/c mice with native MDP1 purified from BCG and screened for Ig-producing B cell hybridoma in lymph nodes and immortal myeloma cells. One isolated hybridoma (7C4C3D4) produced anti-MDP1 IgG, which was designated 7C. We identified that the epitope recognized by mAb7C is located in the 41–60 amino acid region of MDP1, because synthetic peptides corresponding to this region inhibited MDP1-mAb 7C interaction (supplementary Fig. S1A and B). The 41–60 amino acid region of BCG-MDP1 is consistent with that of *M. tuberculosis*-MDP1 (Supplementary Fig. S1 C).

In order to obtain a mAb that recognizes post-translationally modified MDP1 (aforementioned), we synthesized CKKKTKAPAKKAATKKK peptide, whose 10th and 11th Lys are dimethylated (Supplementary Fig. S2A). The peptide's 5th–15th amino acid corresponded to the 141–151 region of *M. tuberculosis*-MDP1 (Supplementary Fig. S2A). The peptide was administered to C57BL/6 mice after conjugation with keyhole limpet hemocyanin (KLH) on its N-terminal Cys residue.

To screen for hybridomas producing antibodies that recognize dimethylated lysin, we synthesized peptides with the same amino acid sequence as immunized peptide, with dimethylation of lysin at the 10th position, at the 11th position, and at both the 10th and 11th positions, and conducted ELISA (Supplementary Fig.S2A and B). We obtained one hybridoma, designated 38–8, produces IgG only recognizes the dimethylation of 10th_,_ 11th or both lysine residues (Supplementary Fig. S2B).

**Figure 1:**
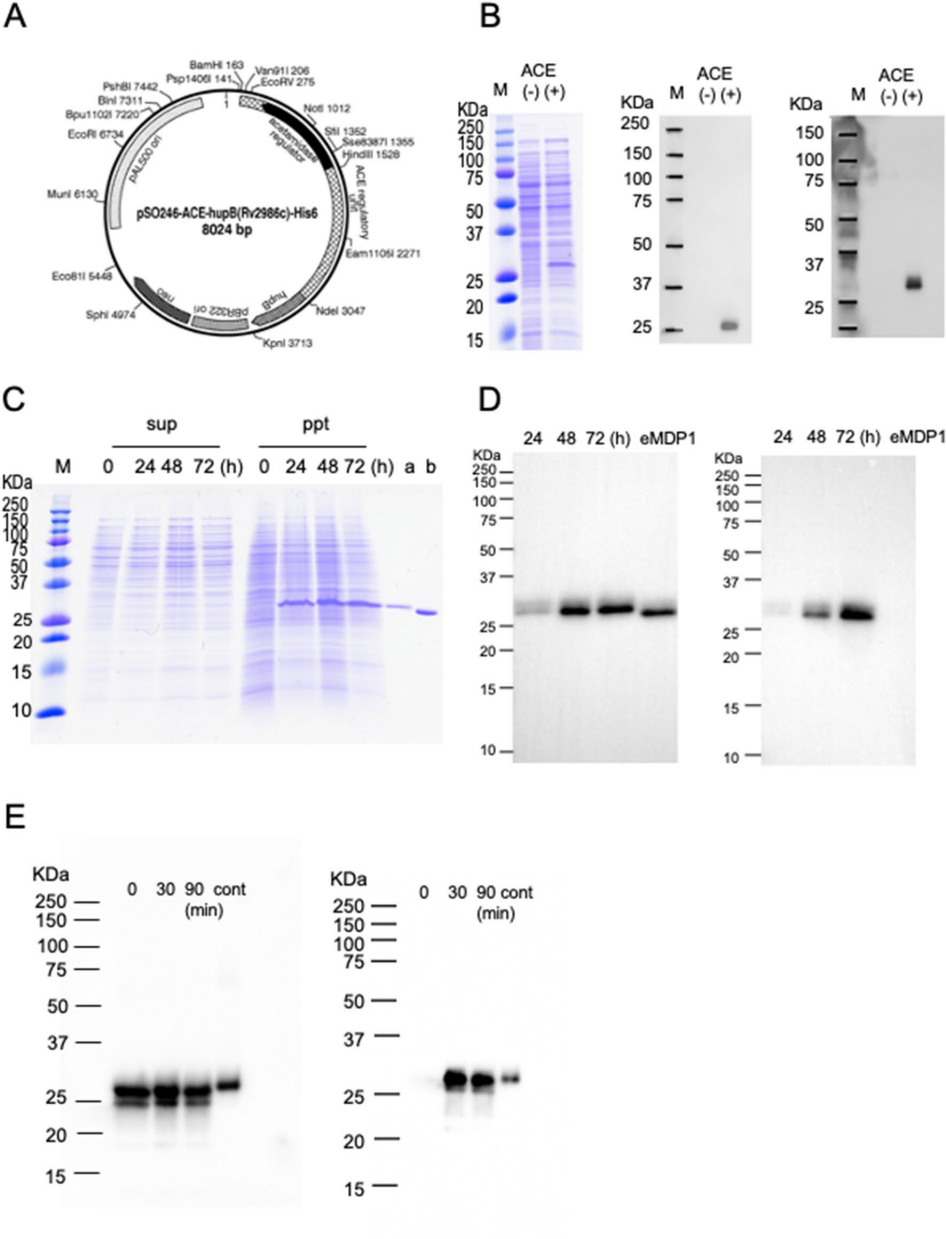
Expression of *M. tuberculosis*-MDP1 in *M. smegmatis*. (**A**) Plasmid map of pSO246-ACE-*hupB-His.* Expression cassette of *M. tuberculosis* MDP1 gene (*hupB*) with His-6 tag driven by an *M. smegmatis* acetamidase promoter was inserted into *E. coli*-mycobacteria shuttle vector pSO246. (**B**) Induction of *M. tuberculosis*-MDP1 expression in pSO246-ACE-*hupB*-His/*M. smegmatis.* pSO246-ACE-*hupB*-His/*M. smegmatis* was cultured for 48 h in the presence or absence of 2% ACE. Ten µg of lysate was fractionated by SDS-PAGE in 12% acrylamide gel and visualized by CBB staining (left). For western blot, 2 µg of bacterial lysate was fractionated and transferred to a membrane, followed by incubation with anti-MDP1 mAb 7C (middle) or anti-His-Tag antibody (right). M: molecular marker, (−): absence of acetamide, ( +): presence of acetamide. (**C**) Time course and soluble fraction of mMDP1. Harvested pSO246-ACE-*hupB*-His/*M. smegmatis* at 0, 24, 48, and 72 h after addition of ACE were treated with bacteria-disrupting agent, BugBuster. Five µg of each soluble (sup) and insoluble (ppt) fraction was fractionated by 12% SDS-PAGE and stained with CBB. a and b, 300 ng of native MDP1 purified from *M. tuberculosis* H37Rv and eMDP1, respectively. (**D**) Detection of MDP1 expression by mAb 7C and mAb 38–8. mMDP1 was purified from pSO246-ACE-*hupB*-His/*M. smegmatis* at 0, 24, 48, and 72 h after addition of ACE. Western blot was then carried out by using mAb 7C (left) and mAb 38–8 (right). One hundred ng of protein and 50 ng of eMDP1 was loaded in each lane. (**E**) Chemical methylation of eMDP1. eMDP1 was treated with methylation reagent for 0, 30, 90 min. Western blot analysis was conducted using an anti-His antibody (left) and mAb38-8 (right). One hundred ng of protein was loaded per lane. cont: mMDP1.

We confirmed that mAb 7C can detect recombinant MDP1 expressed in both *E. coli* (eMDP1) and *M. smegmatis* (mMDP1) by western blot analysis (Fig. 1D, left). In contrast, mAb 38–8 only detected mMDP1 but not eMDP1 (Fig. 1D, right). These data suggest that dimethylation occurred in the 141–151 region of MDP1 expressed in *M. smegmatis* but not in *E. coli*. In addition,

mAb 38–8 also reacted with chemically methylated eMDP1 (Fig. 1E).

We conducted Surface Plasmon Resonance (SPR) analysis to determine the binding kinetics of mAb 7C and 38–8 mAb to MDP1. The dissociation constant (KD) values were 3.08 × 10^–10^ M and 7.56 × 10^–9^ M, respectively (Supplementary Fig. S1D and S2C). These mAbs were utilized for detection of MDP1 in our research.

### Preparation of MDP1 produced in *E. coli* (eMDP1)

Recombinant MDP1 produced in *E. coli* as histidine-tag proteins, which was designated eMDP1, can be purified by previously established methods^13^. However, its purification efficiency was 0.1 mg from 1 L culture of recombinant *E. coli*.

One possible reason for the low yield might be low expression levels due to different codon usage between *E. coli* and *M. tuberculosis*. Another possible reason is unstable mRNA level due to degradation of foreign gene-derived mRNA. As such, we next tested these hypotheses in order to solve the problem of low eMDP1 yield. First, we used a synthetic gene whose codons were optimized to that of *E. coli* (Supplementary Fig. S3). Second, we used Rosetta (DE3) *E. coli* that genetically complemented transfer RNAs of rare codons. In the third option, we used BL21Star (DE3) which stabilizes mRNA. Each recombinant *E. coli* containing MDP1-expression cassette was cultured in LB media and eMDP1 expression was induced by IPTG^13^.

We analyzed eMDP1-expression levels by SDS-PAGE and western blot using mAb 7C or anti-6-HIS antibody. We found elevated eMDP1-expression only when we used Rosetta (DE3) *E. coli* (Supplementary Fig. S4). This suggests that rare codon in *M. tuberculosis*-MDP1 gene can be the bottleneck for MDP1 expression in *E. coli*.

Based on these data, we decided to use Rosetta (DE3) *E. coli* as the host cell for eMDP1 purification. As a result, we could purify 1.0 mg of eMDP1 from 1L culture of Rosetta (DE3) *E. coli.*

### Preparation of MDP1 produced in *M. smegmatis* (mMDP1)

We next evaluated productivity of *M. tuberculosis*-MDP1 in *M. smegmatis*. To achieve that, *M. tuberculosis*-MDP1 with 6-His was inserted into *E. coli-*mycobacteria shuttle vector pSO246 downstream of the acetamidase promoter (pSO246-ACE-*hupB*-His6, Fig. 1A) and electroporated into hygromycin (Hyg) resistant MDP1-deficient *M. smegmatis* (Δ*mdp1*)^12^. This transformant was designated as pSO246-ACE-*hupB*-His/*M. smegmatis*.

To express *M. tuberculosis*-MDP1 in pSO246-ACE-*hupB*-His/*M. smegmatis*, we cultured the transformant in Mueller Hinton II media until optical density at 600 nm (OD_600_) of 1.0 and then added acetamide (ACE), an activator of the acetamidase promoter. Forty-eight hours later, we saw a substantially increased protein band at 28 kDa (Fig. 1B). In the western blot analysis, the same band was recognized by both mAb 7C and mAb against 6X His-Tag (Fig. 1B), showing substantial expression of *M. tuberculosis*-MDP1 by addition of ACE in recombinant *M. smegmatis*.

Next, we searched for optimal time points feasible for mMDP1 purification. mMDP1 was identified in the insoluble fraction of the bacterial lysate following lysis with the BugBuster bacterial lysis reagent (Fig. 1C). The amount of mMDP1 was comparable in lysates obtained at 24, 48, and 72 h after addition of ACE (Fig. 1D, left). We found that mAb 38–8, which recognizes post-translational modifications, reacted with mMDP1. Its reaction level was highest in 72 h after addition of ACE (Fig. 1D, right). However, proteins started degradation probably due to entering the late-stationary phase. We thus chose 48 h incubation with ACE for mMDP1 purification.

We next proceeded with the purification of mMDP1. mMDP1 was not solubilized with commercially available bacterial lysis buffer, the BugBuster reagent (Fig. 1C). Therefore, we employed the acid-extraction method with 0.25 M HCl, which was used to solubilize mycobacterial MDP1^11^. After neutralization, acid solubilized proteins including mMDP1 were denatured by dialyzing in 6 M Urea and applied to a nickel column under the control of AKTA explore FPLC system (Supplementary Fig. S5A). Fractions containing MDP1 were refolded on the nickel column by gradual reducing the Urea concentration from 6 to 0 M (Methods and Supplementary Fig. S5B). We obtained 2.3 mg of mMDP1 from 1 L culture of pSO246-ACE-*hupB*-His/*M. smegmatis.* Thus, the purification efficacy was higher when using *M. smegmatis* as a host cell compared to *E. coli* (1 mg/L).

### Characterization of the secondary structure and oligomerization property of eMDP1 and mMDP1

We showed that both mAb 7C and Anti-6X-His mAb recognized both eMDP1 and mMDP1 (Fig. 1D left). By contrast, mAb 38–8 only recognized mMDP1 (Fig. 1D, right), indicating that mMDP1 contained post-translational modification. We next examined whether post-translational modification may influence the structure of MDP1.

**Figure 2:**
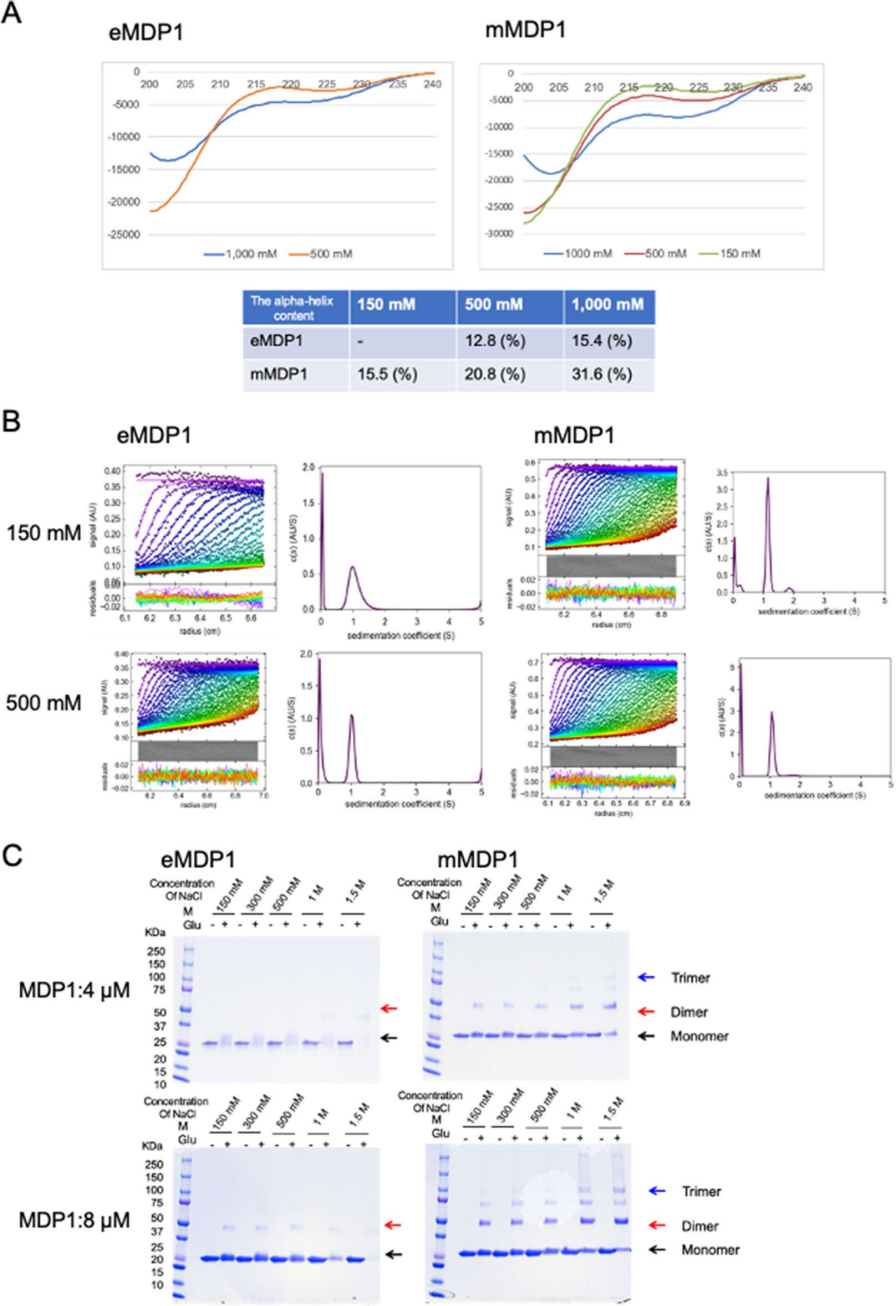
The secondary structure and oligomerization of MDP1. (**A**) CD spectra of eMDP1 and mMDP1. eMDP1 and mMDP1 were adjusted to a concentration of 3.2 μM in 150 mM, 500 mM, and 1000 mM NaCl (pH 7.5). CD spectra of eMDP1 (left) and mMDP1 (right) are shown. The calculated percentage of α helix is indicated in the lower panel. (**B**) Sedimentation velocity measurement of eMDP1 and mMDP1. eMDP1 and mMDP1 were dialyzed against phosphate buffer (pH 7.0) containing 150 mM or 500 mM NaCl before the experiment. Radial fluorescence scans during sedimentation at 20 °C are depicted (dot colors indicate times in the following order: purple-blue-green-yellow–red). Solid lines represent the best-fit with a single species (left). The resulting sedimentation coefficient distributions are shown on the right. The peak of each graph was estimated to represent the monomer. Measurements were conducted with 150 mM NaCl (upper graphs) and 500 mM NaCl (lower graphs) for eMDP1, displayed on the four left columns, and for mMDP1 on the four right columns. (**C**) Glutaraldehyde crosslinking assay of eMDP1 and mMDP1. eMDP1 and mMDP1 in various molar concentrations of NaCl were incubated with or without glutaraldehyde (final concentration 0.2%) at room temperature for 30 min and then applied to SDS-PAGE. Four µM of eMDP1 and mMDP1 was run in the upper gel image, while 8 µM of each was run in the lower gel image. SDS-PAGE was performed by using a 4–15% gradient gel. Gel images on the left column show eMDP1, while those on the right show mMDP1. Monomer (black arrow), Dimer (red arrow), and Trimer (blue arrow) are indicated based on molecular weight.

Circular dichroism (CD) spectra analysis showed that the α-helix content of eMDP1 was not observed at 150 mM NaCl but was 12.8% at 500 mM NaCl and 15.4% at 1000 mM NaCl. In the same experimental setting, the α-helix contents of mMDP1 were 15.5% at 150 mM NaCl, 20.8% at 500 mM NaCl, and 31.6% at 1000 mM NaCl. This suggests that the amount of α-helix increases with the salt concentration, and that the α-helix contents of mMDP1 were around twofold higher than those of eMDP1. This indicates that post-translational modification stabilizes the secondary structure of MDP1 (Fig. 2A).

We next examined the oligomerization status of eMDP1 and mMDP1. Sedimentation velocity (SV) analysis revealed that both eMDP1 and mMDP1 exist as monomers at physiological NaCl concentrations (150 mM), and in 500 mM NaCl (Fig. 2B).

We also performed glutaraldehyde crosslinking analysis to judge the association time between molecules^27^. As expected from the SV measurement data, most eMDP1 and mMDP1 were monomers. Vast majority of eMDP1 were not crosslinked and fractionated as monomers after glutaraldehyde crosslinking, although dimers were detected at 4 µM eMDP1 at 1 M salt concentration and at 8 µM eMDP1 at all salt concentrations (Fig. 2 C left panels). In contrast, part of mMDP1 was crosslinked at all tested salt concentration, and dimeric and trimeric crosslinked forms increased at higher salt concentrations (Fig. 2C right panels). The glutaraldehyde crosslinking study suggests that the association time between proteins was longer for mMDP1 than for eMDP1.

Taken together these results indicate that one, most eMDP1 and mMDP1 are monomers at physical condition, two, the dimer/monomer ratio increase in the salt concentration for both eMDP1 and mMDP1, and three, mMDP1 tends to oligomerize more frequently than eMDP1.

### mMDP1 stimulates IFN-gamma production by peripheral blood cells derived from BCG-vaccinated individuals

Th1-type cells produce IFN-gamma which is critical for vaccine-induced protection against tuberculosis both in mice and human^25,26^. To assess the feasibility of eMDP1 and mMDP1 as booster vaccine antigens, we next evaluated their ability to stimulate IFN-gamma production by peripheral blood mononuclear cells (PBMC) or whole blood cells derived from BCG-vaccinated individuals. The selected donors received BCG (BCG Tokyo 172) vaccine over 20 years ago, and it is believed that the efficacy of BCG has since diminished^28^.

**Figure 3:**
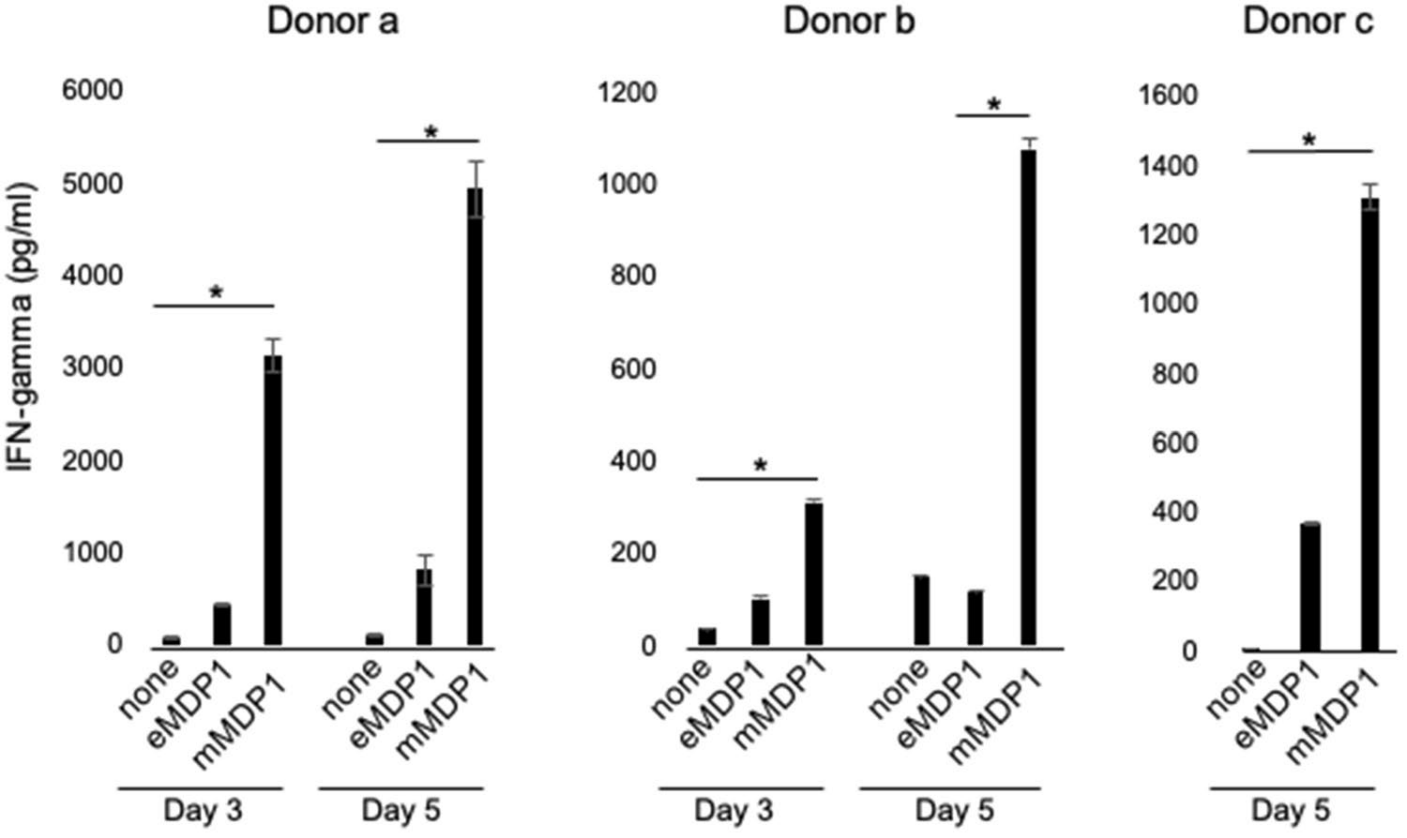
IFN-gamma production induced by ex vivo stimulation of PBMC with eMDP1 or mMDP1. Peripheral blood mononuclear cells (PBMC) derived from three donors were plated at concentration of 2 × 10^5^ cells per well and incubated with either 0.5 μM of eMDP1 or mMDP1, or untreated (none). The IFN-gamma production level of three days after culturing of two donors and five days after culturing of three donors is presented in the mean ± SD. *P < 0.05. Data were analyzed using Friedman tests followed by Dunn’s multiple comparison test. Each bar represents the mean of triplicate values. Error bars, SD of the means. The results shown are representative of two experiments.

PBMC from 3 individuals was cultured with either eMDP1 or mMDP1 followed by quantification of IFN-gamma levels in culture supernatants. We found that all tested PBMC from 3 individuals produced higher amounts of IFN-gamma when stimulated by mMDP1 than by eMDP1 (Fig. 3). These data show stronger induction of IFN-gamma production by mMDP1 than eMDP1 in BCG-vaccinated individuals.

### Comparison of immuno-stimulatory efficacy of MDP1 to other major *M. tuberculosis* antigens

Several immunodominant proteins are proposed vaccine candidates against tuberculosis^29^. More attention is focused on secreted proteins as they are likely to efficiently stimulate immune cells compared to non-secreted proteins^30^. We therefore evaluated the immunogenicity of mMDP1 to comparable to that of secreted *M. tuberculosis* proteins.

For this purpose, we purified secreted *M. tuberculosis* proteins from *M. tuberculosis* culture filtrates according to previous established methods^31^. The purified proteins were identified as MPT 32 (Rv1860, or Apa), MPT 45 (Rv0129c, Antigen 85C, or fbpC), MPT 46 (Rv3914, TrxC), MPT53 (Rv2878c, or mpt53), MPT 59 (Rv1886c, Antigen 85B, or fbpB), and MPT 63 (Rv1926c, mpt63) by mass spectrometry^32^.

**Figure 4:**
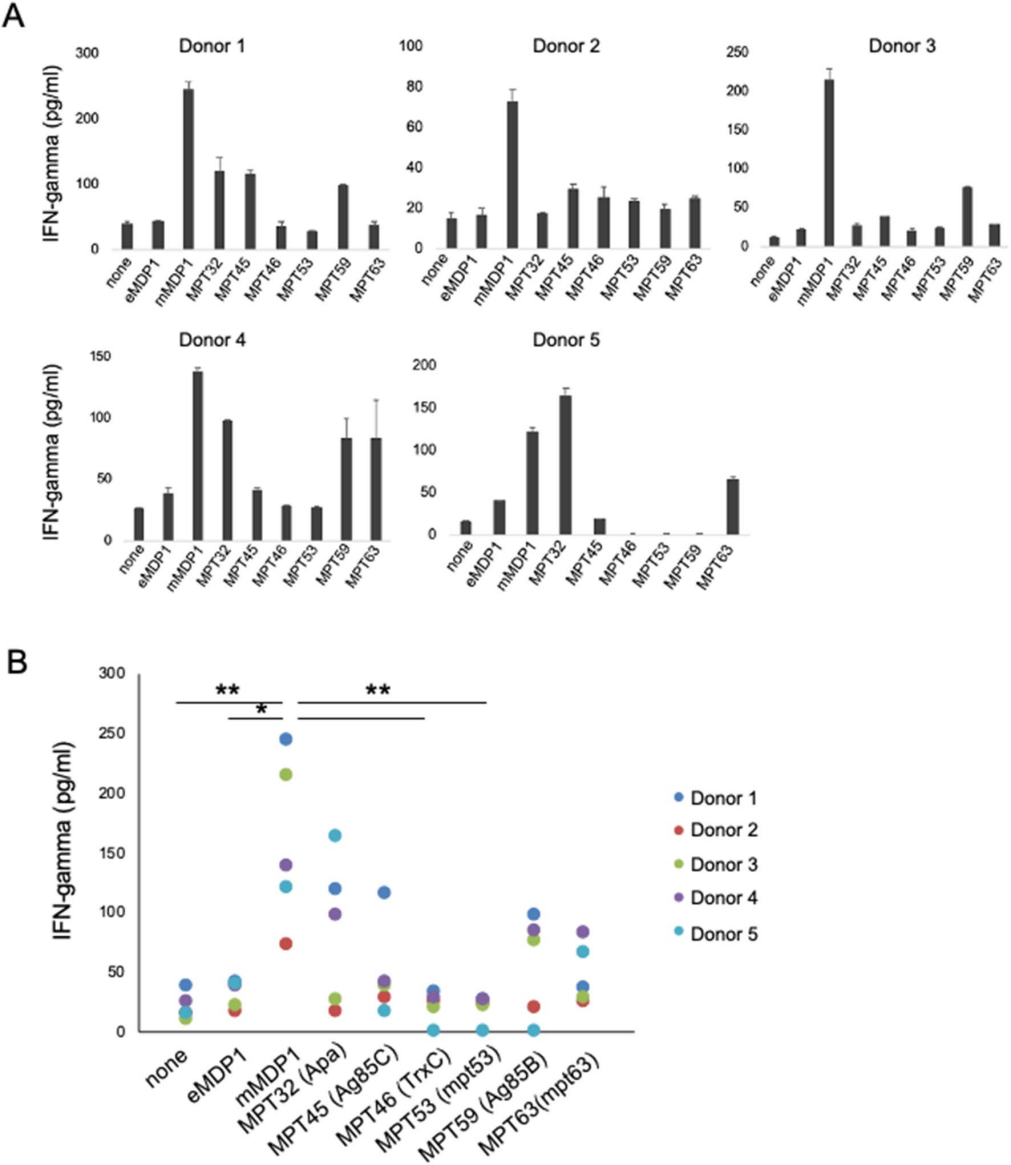
IFN-gamma productivity of mMDP1 and other *M. tuberculosis* proteins in BCG-vaccinated individuals. (**A**) Whole bloods samples obtained from five donors were incubated with 0.5 µM eMDP1, mMDP1 or, other *M. tuberculosis* antigens, including vaccine candidates such as MPT32, MPT45, MPT46, MPT53, MPT59, MPT63. After three days of cultivation, the concentration of IFN-gamma in culture supernatant was quantified using ELISA. Each bar represents the mean of triplicate values. Error bars, SD of the means. (**B**) The combined results of five donors are presented. Statistical significance: *, *p* < 0.05; mMDP1 versus none, eMDP1, MPT 46, and MPT 53, respectively. Data were analyzed using Friedman tests followed by Dunn’s multiple comparison test. The results shown are representative of two experiments.

We incubated each purified secreted protein or mMDP1 with bloods derived from 5 healthy individuals who were vaccinated with BCG more than 20 years ago, and measured IFN-gamma production. We found heterologous IFN-gamma production among donors (Fig. 4A). However, the level of IFN-gamma production in blood stimulated with mMDP1 was higher than when stimulated with any secreted proteins including immunodominant proteins such as MPT32, antigen 85B (MPT59) and C (MPT45), except in the case of MPT32 for 1 donor (Fig. 4A). These data suggest that mMDP1 is more immunogenic than secreted *M. tuberculosis* proteins in BCG-vaccinated individuals (Fig. 4B).

### Determination of mMDP1 post- translational modification

**Figure 5:**
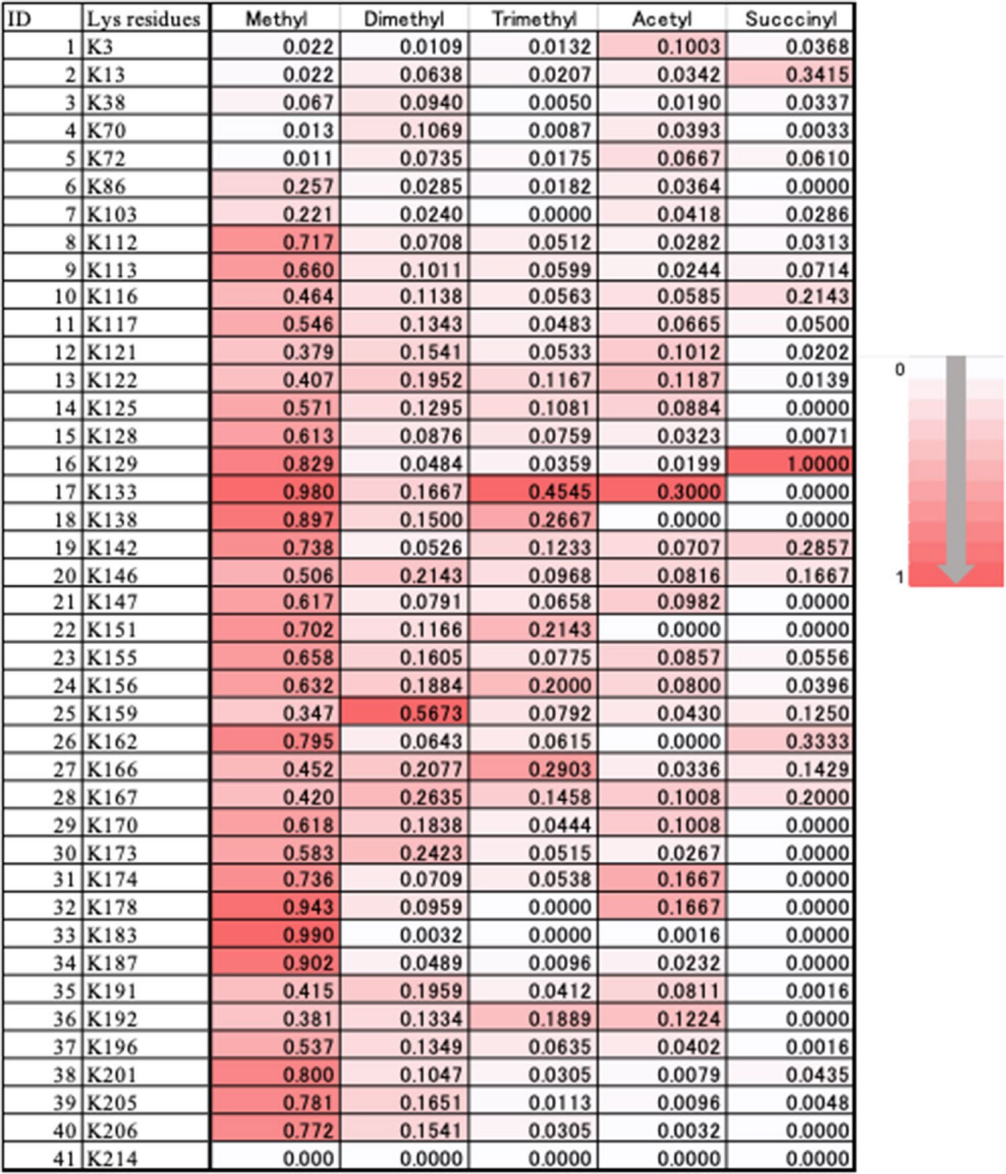
Post-translational modifications on mMDP1 lysine residues. Peptides generated by limited proteolysis of mMDP1 with trypsin were analyzed by mass spectrometry to determine the extent and localization of post-translational modifications. This included methylation, dimethylation, trimethylation, acetylation, and succinylation on all MDP1 lysine residues. The frequencies of each post-translational modification on lysin residues are depicted as heat maps. The values within each cell represent the ratio of post-translationally modified lysine to total lysine.

Our data shows that mMDP1 is more immunogenic than eMDP1. This could be due to post-translational modifications in mMDP1, which are absent in eMDP1. Although mAb 38–8 recognizes mMDP1 but not eMDP1 (Fig. 1D, right), the entire post-translational modifications on mMDP1 are unknown. We next identified post-translational modifications in mMDP1 by utilizing a newly developed method using limited proteolysis with trypsin in combination of liquid chromatography-electrospray tandem mass spectrometry (LC/MS)^33^. The data shows that, compared to eMDP1, most of the lysine residues on the C-terminal half of mMDP1 were highly methylated, moderately dimethylated, trimethylated, and acetylated^34^ (Fig. 5). Importantly, the pattern of post-translational modifications in mMDP1 closely resemble that of wild-type *M. tuberculosis* MDP1^33^, which might explain high antigenicity of mMDP1 in BCG-vaccinated individuals.

### Evaluation of adequate CpG-ODN adjuvant for MDP1

Synthetic oligonucleotides containing CpG motifs (CpG ODN) are considered ideal adjuvants for MDP1, since they increase immunogenicity of MDP1^11^. We next sort to determine the suitable CpG ODN to increase the antigenicity of MDP1. CpG-DNAs are categorized into 3 classes based on their structure. We used ODN 2216 (Class A), ODN 2006 (Class B), and ODN 2395 (Class C) in this study. In addition to these representative CpG-DNAs, we used a new class of CpG-ODN designated as G9.1, that we recently developed^23^ (Supplementary Table 1). As control, we used their negative sequence forms–CpG motif was changed to GpC–as control (Supplementary Table 1).

**Figure 6:**
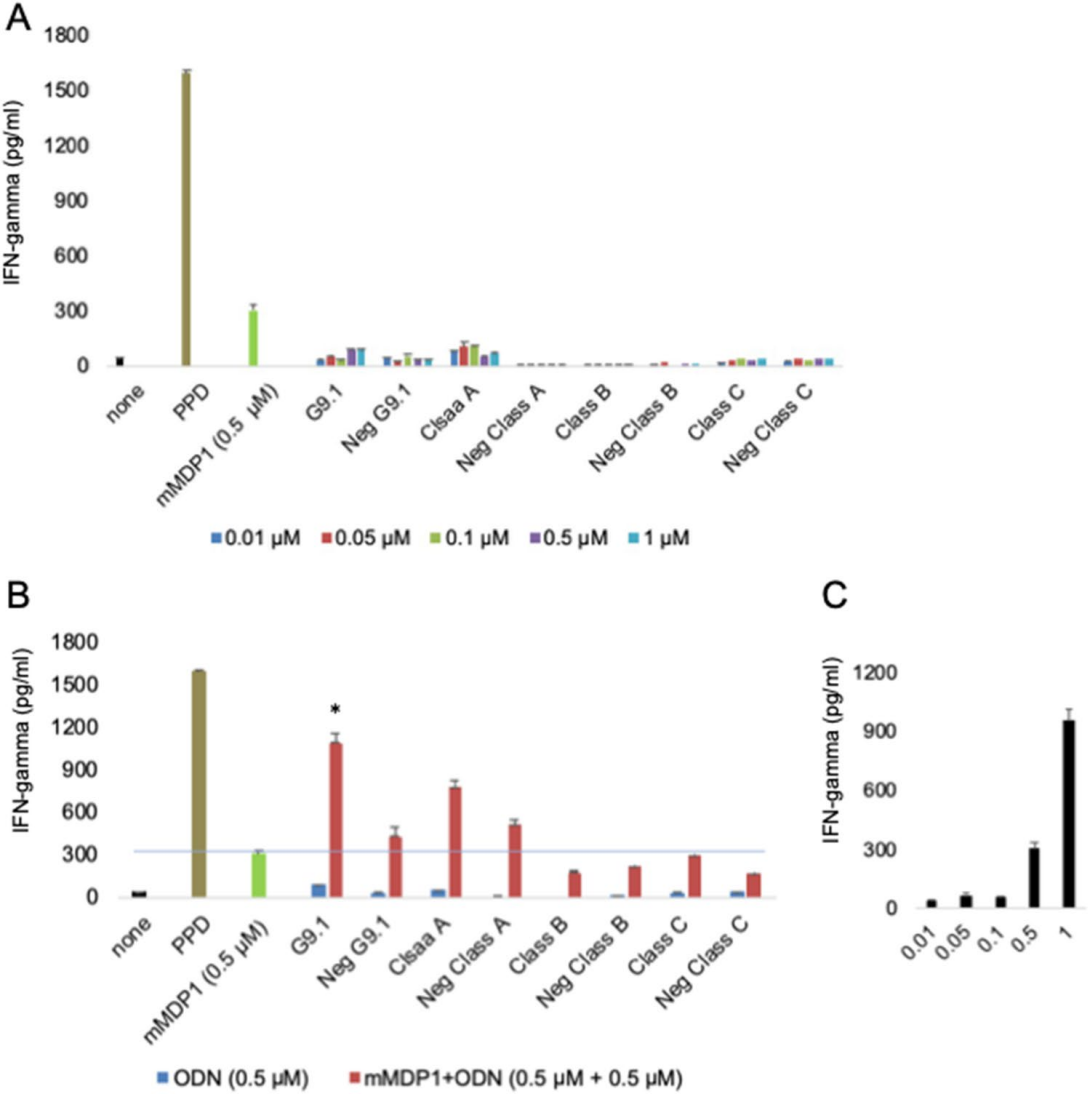
Evaluation of CpG ODNs as adjuvants for mMDP1. Whole blood from a donor was diluted with an equal volume of RPMI and supplemented with IL-2 (20 U/ml). The culture was conducted with 0.01, 0.05, 0.1, 0.5, and 1 μM of phosphodiester bonded ODNs either alone or in combination with mMDP1 (0.5 μM) for 3 days. The amount of released IFN-gamma was measured using ELISA. (**A**) IFN-gamma production when blood was cultured with ODNs alone (0.01 ~ 1 µM). (**B**) IFN-gamma production when whole blood was cultured with 0.5 μM ODNs only (blue columns) or in combination with mMDP1 (0.5 μM) (red columns). The blue line and green column represent IFN-gamma production when blood was cultured with 0.5 μM of mMDP1 alone. *, *P* < 0.05, indicating the comparison of IFN-gamma production stimulated by 0.5 µM of mMDP1 alone versus in combination of ODN + 0.5 µM mMDP1. Data were analyzed using Friedman tests followed by Dunn’s multiple comparison test. (C) IFN-gamma production when blood was cultured with mMDP1 only (0.01 μM ~ 1 μM). Each bar represents the mean of triplicate values. Error bars, SD of the means. The results shown are representative of two experiments.

We measured IFN-gamma production from whole blood sample obtained from a healthy BCG-vaccinated individual after stimulation with mMDP1 or a combination of mMDP1 and each ODN as adjuvant (Fig. 6). ODNs at concentrations ranging from 0.01 M to 1 µM induced little IFN-gamma production (Fig. 6A). When blood was cultured with 0.5 µM of mMDP1 alone, 302 ± 29.9 pg/ml of IFN-gamma production was observed (Fig. 6C). Combination of mMDP1 with either Class B, Negative form (Neg) Class B, Class C, or Neg Class C did not exceed stimulatory effects by mMDP1 alone. In contrast, the combination of mMDP1 with G9.1 or ODN 2216 (Class A) produced more IFN-gamma than mMDP1 alone. The highest IFN-gamma production was observed by the combination of mMDP1 and G9.1 (1180 ± 258 pg/ml) (Fig. 6B).

**Figure 7:**
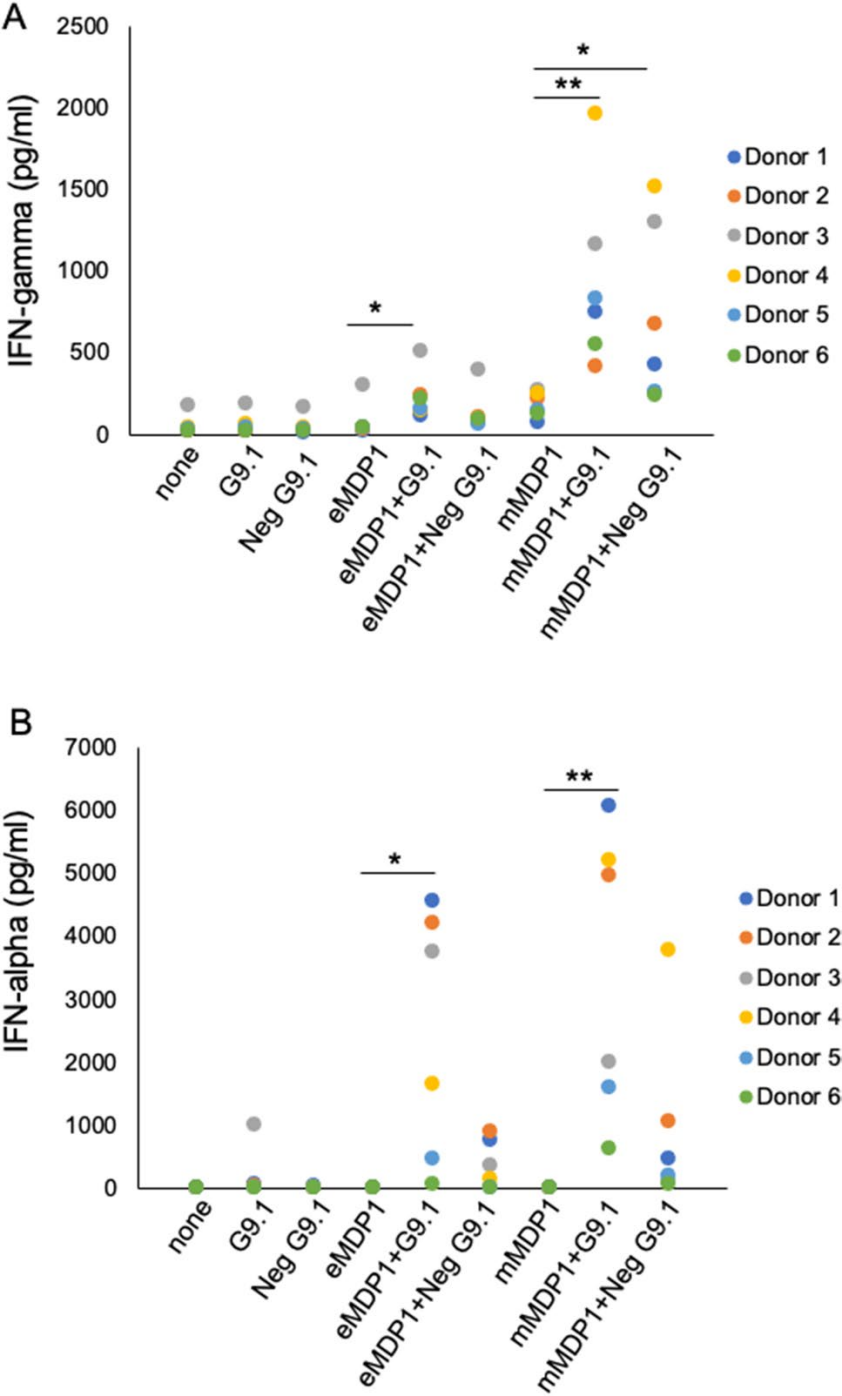
Augmented production of IFN-gamma and IFN-alpha when blood was stimulated with MDP1 and G9.1. Whole blood derived from six donors was cultured with MDP1 alone, or in combination with G9.1, or in combination with the negative form of G9.1(Neg G9.1). After three days of incubation with IL-2 at a concentration of 20 U/ml, the concentration of IFN-gamma (**A**) and IFN-alpha (**B**) in the culture supernatant were measured using ELISA. Statistical significance: *, *p* < 0.05. Data were analyzed using Friedman tests followed by Dunn’s multiple comparison test. The results shown are representative of two experiments.

We increased the number of BCG-vaccinated donors to confirm augmentation of MDP1-induced IFN-gamma production by G9.1 (Fig. 7A). The combination of mMDP1 and G9.1 induced significantly higher IFN-gamma production than mMDP1 alone, and the combination of eMDP1 and G9.1. Unexpectedly, combination of mMDP1 and Neg G9.1, non CpG-DNA also induced more IFN-gamma production than mMDP1 alone (Fig. 7A).

It is reported that, in human peripheral blood cell populations, B cells, monocytes, and plasmacytoid dendritic cells (pDC) expresses TLR9, the non-methylated CpG-DNA receptor^20,35,36^. Among these cells, pDC produces massive amount of IFN-alpha in response to CpG-ODNs^37^. As such, we next tested IFN-alpha production from blood cells in the same experimental setting. We observed remarkable production of IFN-alpha when blood samples were stimulated with a combination of eMDP1 or mMDP1 and G9.1. However, mMDP1 exhibited a tendency to be more immunogenic. In contrast, IFN-alpha production when eMDP1 or mMDP1 was combined with Neg G9.1 resulted in lower IFN-alpha production compared to the combination with G9.1. This suggests that TLR9 stimulation is required for IFN-alpha production (Fig. 7B). These results show the importance of TLR-9 for IFN-alpha production by pDC stimulated with MDP1-G9.1 complex.

### mMDP1 binds more strongly to G9.1 than Class A, Class B, and Class C ODNs

We observed synergistic effects on immune-stimulation by combination of mMDP1 with G9.1, Neg G9.1, or ODN 2216 but not with other tested CpG-ODNs. Since MDP1 is a DNA-binding protein, we examined whether mMDP1 binds directly to each CpG-ODN using Bio-Layer Interferometry.

We found that mMDP1 bound to G9.1 and Neg. G9.1 more strongly than ODN 2216 (Class A) and ODN 2006 (Class B). mMDP1 did not bind to ODN 2395 (Class C). The affinity of mMDP1 with G9.1 and Neg G9.1 were similar and calculated to be 6.73 × 10^–9^ M, 4.93 × 10^–9^ M. On the other hand, the affinity of mMDP1 with Class A (2.50 × 10^–8^ M) and Class B (5.83 × 10^–8^ M) were lower (Supplementary table 2). These results suggest that the heightened levels of IFN-gamma production by immune cells stimulated by the combination of mMDP1 and CpG-DNAs are correlated with their mutual affinity.

### G9.1 promotes binding of MDP1 to PBMC

Binding to cell surface receptors is the initial step for immune activations by ligands. In order to identify the mechanism of synergistic immune-stimulation by mMDP1 and G9.1, we evaluated interaction of mMDP1, G9.1, and mMDP1- G9.1 mixture with PBMC by flow cytometry analysis.

**Figure 8:**
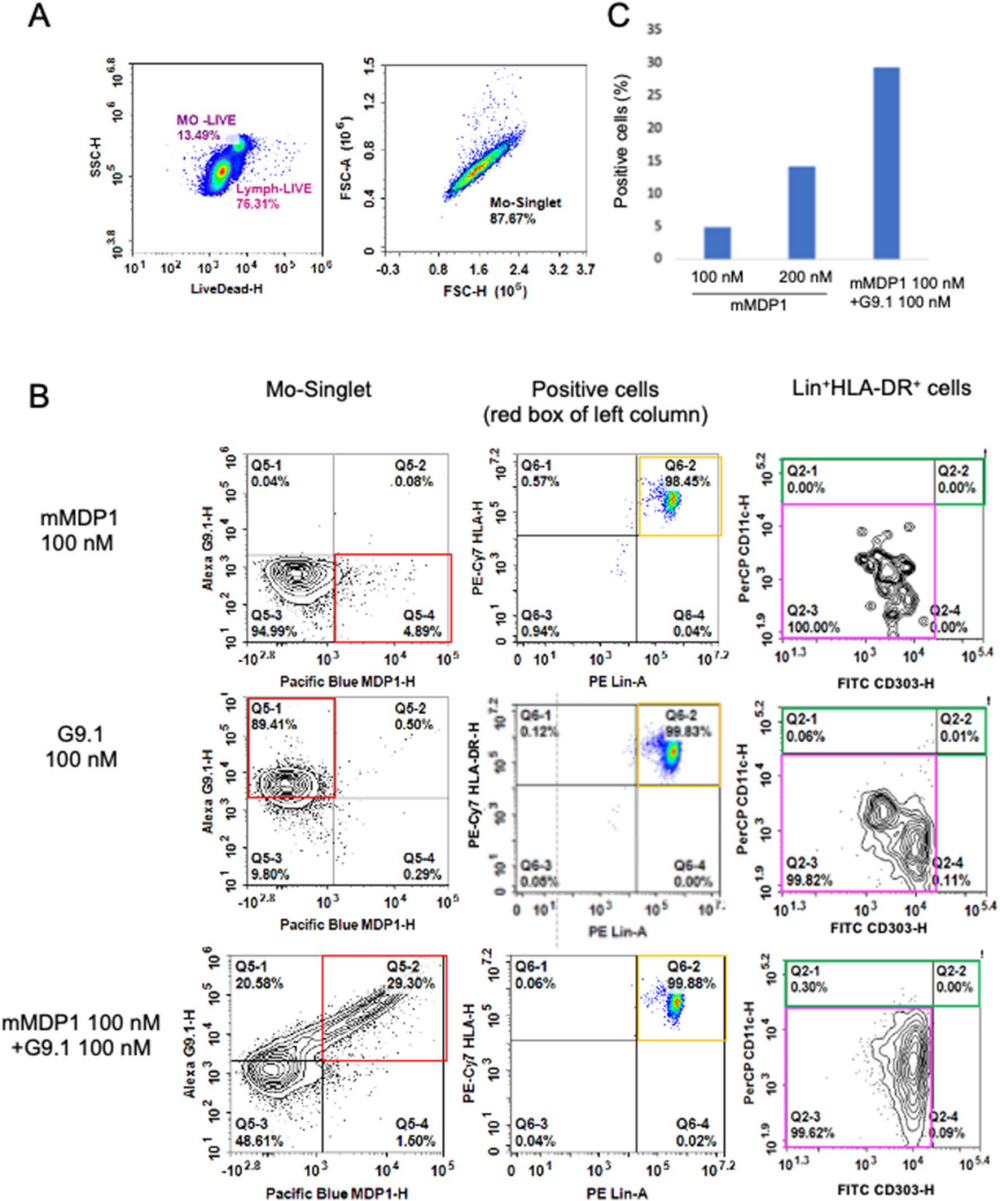
mMDP1 and G9.1 binding to immune cells populations. PBMCs (1 × 10^6^ ~ 2 × 10^6^/ml) derived from a donor were cultured with Pacific Blue labeled mMDP1, Cy5 labeled G9.1 or a mixture of mMDP1 and G9.1 for 1 h at 5% CO2 and 37 °C. Next, the cell surface was stained with antibodies according to manufacturer’s protocol and fixed with 4% paraformaldehyde in staining medium overnight. Cells were then analyzed the following day using the flow cytometer, NovoCyte 3000. (**A**) Analysis of the total cell population (left panel) and the monocyte-singlet region (right panel). (**B**) Cells that are positive for only mMDP1, G9.1, and both mMDP1 and G9.1 is highlighted with a red box (left column). These cells in the figure label “red box from the left column” were Lineage positive and HLA-DR positive antigen presenting cells (orange box in middle column), but negative for CD11c and CD303(pink box in right column). The green box indicates the dendritic cell region (right column). (**C**) Percentage of cells that are positive for mMDP1 or both mMDP1 and G9.1 in monocyte-singlet region (red box in left panel of B). The results shown are representative of two experiments.

We found that majority of MDP1, G9.1, and mMDP1- G9.1 mixture bound to the cells in the lineage plus and HLA-DR plus region, which mediates antigen presentation to T cells in the periphery rather than dendritic cells (Fig. 8B, middle and right column). The percentage of cells bound to mMDP1 increased with the addition of G9.1 compared to mMDP1 alone (Fig. 8C). These results indicate that mMDP1-G9.1 complex can more efficiently access antigen presenting cells compared to its individual components. This finding partially explains their synergistic immune stimulations of human PBMC.

## Discussion

BCG, the tuberculosis vaccine, is administered from the neonatal period to early childhood. While it is effective against tuberculosis in childhood, its effectiveness gradually wanes with age. This study aimed to develop a booster tuberculosis vaccine for individuals who have already been BCG-vaccinated. MDP1 is a major *M. tuberculosis* protein and a candidate for booster vaccine component^22,38^. Here we evaluated its production, antigenicity, immunogenicity, and optimized its accompanying adjuvant.

We first discovered that using Rosette (DE3) *E. coli* supplemented with tRNAs for AGG, AGA, AUA, CUA, CCC, and GGA codons increased MDP1 expression. This suggest that for *E. coli*, MDP1 contains rare codons which can partly explain its low expression in *E. coli* (Figs S3, and S4). We next observed that larger amounts of MDP1 could be purified from *M. smegmatis* compared to Rosette (DE3) *E. coli*. This suggests that, for expression of MDP1, the intracellular environment in *M. smegmatis* is more suitable than that in *E. coli*. These findings highlight the merit of using *M. smegmatis* not only for the expression of MDP1 but also for other mycobacterial proteins.

Monoclonal antibody 38–8 (mAb 38–8), which reacts to the 146th and 147th dimethylated lysins of MDP1, recognizes mMDP1 expressed in *M. smegmatis*, indicating that these two lysines are dimethylated (Fig. 1D and S Fig. 2B). *M. tuberculosis* MDP1 contains as many as 41 residues of lysin, but the details of their post-translational modification have not been revealed due to its complexity, which resists conventional analysis, although only acetylation of MDP1 was reported^14^. We recently established a method to determine multiple post-translational modifications on a protein^33^. By employing this method, we revealed that both native MDP1 (nMDP1) and mMDP1 exhibit highly similar lysin methylation patterns at the C-terminal regions^33^. Although the methyltransferase responsible for MDP1 methylation has not yet been identified, these results suggest that the methyltransferases involved in the methylation of lysine residues on MDP1 in both *M. tuberculosis* and *M. smegmatis* share the same or very similar substrate specificity^17^. This implies that *M. smegmatis* can produce MDP1 with the same physical characteristics as that produced by *M. tuberculosis*.

We acknowledge some limitations in our study. Firstly, The number of participants is small because of single center study. Second, diagnosis of HIV infection nor QFT test to clarify latent tuberculosis infection (LTBI) was not conducted. Thirdly, the infection with NTM was not confirmed through antibody testing. However, we found that post-translationally modified mMDP1 has much stronger immunogenicity than eMDP1. mMDP1 elicits a larger production of IFN-gamma by PBMCs derived from BCG-vaccinated individuals (Figs. 3 and 7). The immunogenicity of mMDP1 is comparable to or greater than that of the tested secreted proteins (Fig. 4). This may be because some MDP1 is also present on the cell wall, where it is easily recognized by immune cells^39^. This strong mMDP1 immunogenicity in BCG-vaccinated individuals indicates its potential use as a booster vaccine antigen.

MDP1 post-translational modifications produced by BCG have not been analyzed, but in view of the concordance between the modification patterns of mMDP1 and nMDP1, it is likely that similar modifications are occurred on MDP1 produced by BCG. Cells that produced IFN-gamma in response to mMDP1 stimulation (Figs. 3 and 7) are thought to be T cells that were activated after donors were vaccinated with BCG. Therefore, the heightened immunogenicity of mMDP1 compared to eMDP1 in donors is likely attributed to IFN-gamma-producing T cells recognizing the post-translationally modified MDP1 produced by BCG. Additionally, we successfully established mAb 38–8, which specifically recognizes post-translationally modified MDP1. Thus, this study confirms that both T and B cells can accurately recognize the post-translationally modified polypeptides.

Post-translational modifications alter or confer functions of proteins and, in some cases, stabilize the protein structure. They also mark proteins for degradations. In this way, there are many proteins that are post-translationally modified in living organisms. On the other hand, immune responses specifically recognize the antigens of pathogens with accuracy. Therefore, post-translationally modified polypeptides are also targets for recognition by immune responses.

Besides HBHA and MDP1, there have been reported to exhibit post-translationally modified proteins in *M. tuberculosis*. Thus, ESAT6 is acetylated, Apa/Rv1860/MPT32 and PstS1 are O-glycosilated protein, and LpqH is lipoproteins^40^. Interestingly all of these post-translationally modified proteins show strong immunogenicity. ESAT6 is a major virulence factor of *M. tuberculosis*, which is lacking in an attenuated strain, BCG. Acetylation of Ser and Thr resides in ESAT6 has been documented^41^. Interestingly, native ESAT6 from *M. tuberculosis* showed efficacy comparable to BCG against *M. tuberculosis* infection, whereas rESAT6 produced in *E. coli* showed no protective effect^42^. Apa is a mannosylated protein^40^_._ Stimulation with mannosylated native Apa has been shown to enhance the production of cytokines such as IL-4, IL-17, and IFN-gamma from BCG vaccinated human PBMCs and spleen or lung cells of BCG-infected mice^43^. This mannosylated Apa-peptide was indeed recognized by an established HLA-E-specific human CD8^+^ T cell^44^. LpqH lipoprotein is recognized through toll-like receptor 2 and triggers Th1 responses^45^. PstS1 was observed the immunodominant 38 kDa protein^46^ and a recent report suggested anti-PstS1 antibodies contribute to host protection in humans^47^. We recently reported antibody levels against native *M. tuberculosis* proteins including Apa and PstS1, but the corresponding *E. coli* recombinant proteins were unable to detect pulmonary tuberculosis patients^32^. Exact reasons for the strong immunogenicity of post-translationally modified proteins of *M. tuberculosis* can be complex, but this is fundamentally due to accuracy of immune responses. Thus, taking into consideration of the native structure of antigens is important for understanding immune responses and developing relevant attractive strategies, such as, vaccines and immunotherapies.

MDP1 and CpG-DNA synergistically enhance each other's immunogenicity^22^_._ Therefore, to utilize mMDP1 as a vaccine component, we examined the adjuvant effects of type A–C and G9.1 CpG- DNA. The results showed that G9.1 strongly enhanced the immunogenicity of mMDP1. This enhancement was attributed to the strength of their binding, as observed in the analysis using Bio-Layer Interference (Sup Table 2). These results suggest that the immunogenic enhancement of MDP1 by CpG-DNA depends on their affinity. This is reasonable, as MDP1 can be efficiently presented by antigen-presenting cells activated by the CpG-DNA simultaneously. Indeed, the primary cells taking up mMDP1 were the HLA-DR positive cell population, which possesses antigen-presenting capacity, and the uptake rate was further increased by the addition of G9.1 (Fig. 8). This may explain the synergistic immune activation of MDP1 and G9.1.

The results of this study demonstrate important implications for the development of component vaccines. Specifically, structural information of antigens produced by pathogens should be considered in vaccine design. This is because proteins from live vaccines and pathogens may have unique post-translational modifications, which are well recognized by the host’s immune system^16^. In the development of viral vector or mRNA vaccines using *M. tuberculosis* antigens, the specific post-translational modifications of *M. tuberculosis* are not taken into account. In such cases, the structure of the antigen produced by the vaccine may differ, even if the amino acid sequence is identical.

In this study, we investigated the potential of MDP1 as a booster vaccine, primarily focusing on its ability to enhance IFN-gamma production. We specifically explored the immunogenicity of MDP1 produced by *M. smegmatis*(mMDP1), in combination with G9.1-CpG-DNA. This combination showed promise in stimulating IFN-gamma production from immune cells in BCG-vaccinated individuals, although we didn’t quantify the number of IFN-gamma producing cells using flow cytometry or ELISPOT assays. IFN-gamma is a crucial cytokine in the defense against tuberculosis (TB), and its production is considered vital for an effective TB vaccine^24–26^. However, it’s important to note that excessive production of IFN-gamma can lead to tissue destruction^48,49^. Moreover, relying solely on IFN-gamma production as a marker of vaccine efficacy may not be sufficient. Recent reports have highlighted the importance of considering non-Th1 cytokines^50^, antibody levels^51^, and innate immune activation at the time of vaccination^52^ as additional indicators of vaccine efficacy. While the mMDP1 and G9.1 combination appears promising as a booster vaccine for TB, it is imperative to investigate other markers beyond IFN-gamma productivity. In addition, we’ve not evaluated real efficacy of booster vaccination of mMDP1 and G9.1 in animal models and BCG-primmed individuals. We previously reported that even eMDP1 plus G9.1 booster immunization was effective against *M. tuberculosis* challenge in BCG-primed guinea pig^22^. It is reasonable to next investigate whether mMDP1 is superior to eMDP1 in BCG-primed guinea pig model before starting clinical study.

## Methods

### Bacterial strains, culture media, and reagents

MDP1 deficient *M. smegmatis* (Δ*mdp1*) was kindly provided by Dr. John L. Dahl (University of Minnesota Duluth). *E. coli* DH5α strain was used for all gene manipulations and was cultured in LB broth or on LB agar (both from Sigma-Aldrich, St. Louis, MO). Rosetta2 (DE3) pLyS (Novagen, Germany), BL21 Star (DE3) pLyS, and BL21Star (DE3) (invitrogen, CA), were also cultured in LB broth to obtain recombinant MDP1. *M. smegmatis* strain was cultured in Mueller HintonII media (BD, MD) supplemented with 0.05% (v/v) Tween 80, 50 μg/ml Hygromycin B (Hyg), and 10 μg/ml kanamycin (Km).

Hyg, Km, chloramphenicol, and isopropyl b-D-thiogalactopyranoside (IPTG) were purchased from FUJIFILM Wako Pure Chemical cooperation (Osaka, Japan). Carbenicillin was purchased from Nacalai tesque (Kyoto, Japan). Acetamide was purchased from Sigma-Aldrich (St. Louis. MO).

### Establishment of anti-MDP1 antibody (7C) and identification of its recognition site

BALB/c mice were immunized with MDP1 extracted from *Mycobacterium tuberculosis* var. Bacille Calmette-Guérin (BCG), and Ig-producing B cells obtained in lymph nodes was fused with an immortal myeloma cell. One isolated hybridoma (7C4C3D4) produced anti-MDP1 IgG, designated 7C. Hundred µl of MDP1(5 μg/ml) was immobilized on Nunc MaxiSorp™ Immuno Plate 1 overnight at 4 °C. After washing with PBS containing 0.05% Tween 20, the plate was incubated with a mixture of 500 ng/ml of overlapped peptide (P1 ~ P21, Supplementary Fig. 1A) and 5 ng/ml of 7C. After 1 h incubation, the plates were treated with a secondary antibody (HRP conjugated Polyclonal Rabbit anti-mouse Immunoglobulins, DAKO, P0260). The reaction was developed with KPL Sure Blue Reserve TMB Microwell Peroxidase Substrate (Sera Care, 5120-0083), recognition site was determined by reduction of absorbance of OD450 nm. Binding potency of MDP1 and 7C was measured by Biacore (Cytiva, Mariborough, MA). MDP1 purified BCG (4846 ng, 261 ng, and 140 ng) was immobilized on CM5 chip, and then reacted with 7C (1.06 nM ~ 68 nM) at 20 µl/min flow rate. KD value was calculated using the manufacturer’s provided software.

### Development of mAb 38–8 which recognizes mMDP1 post-translational modification

C57BL/6NCrSlc mice was immunized via tail base with approximately 85 µg of dimethylated peptide of the 10th and 11th lysine (CKKKTKAPAK (10)K(11) AATKKK) with Freund's Incomplete adjuvant. Two weeks later, the mice were additionally immunized with 125 µg of KLH-crosslinked conjugated sample via tail base. Three days after the additional immunization (17 days after the initial immunization), cells were harvested from ilioinguinal lymph nodes and then, fused with myeloma SP2 cells. To examine m38-8 recognition sites, ELISA test was performed by using four different peptides (dimethylated 10th lysine, dimethylated 11th lysine, both 10 and 11 dimethylated, and unmodified) conjugated with BSA. These were plated on flat-bottom plate (3 µg/ml, 50 µl/well), and ELISA tests were performed with the addition of hybridoma culture supernatant. Affinity of dimethylated mMDP1 and m38-8 was measured by Biacore. The measurement method was the same as 7C.

### Inducible expression of Mtb MDP1 (mMDP1) in *Mycolicibacterium smegmatis*

The construction of inducibly expressing *Mycobacterium tuberculosis* (Mtb) MDP1 was conducted as previously descrived^12^. Breifly, sequences encoding MDP1 (*hupB*, Rv2986c) was amplified using *Mycobacterium tuberculosis* DNA as a template, incorporating HindIII and Nde1 restriction enzyme sites at the N-terminus and adding 6 × histidine tag and KpnI restriction enzyme site at the C-terminus. The amplified DNA fragments (HindIII-NdeI-*hupB*-His6-Kpn1) was excised with Kpn1 and HindIII, and then inserted between Kpn1 and HindIII sites of *Echerichia coli-*mycobacteria shuttle vector pSO246 (Km^R^). The constructed plasmid was designated as pSO246-*hupB-His6.* DNA cassette (ACE unit) which involved the loci from acetamidase regulator gene (accession No. U63095) and acetamidase promoter region (accession No. X57175) in pCRII-TOPO was excised with BamH1 and Nde1. ACE unit was inserted in pSO246-*hupB-His6* which was excised with BamH1 and Nde1 (pSO264-ACE-*hupB-His6*). The self MDP1 deficient *M. smegmais* [Δ*mdp1* (Hyg^R^)] was then transformed with the constructed plasmid (pSO264-ACE-*hupB-His6*) and transformants selected on the 7H11-OADC agar plates containing 50 μg/ml Hyg and 10 μg/ml Km.

*M. smegmatis* inducibly expressing Mtb MDP1 (pSO246-ACE-*hupB*-His/*M. smegmatis*) was grown in Sakaguchi flask with Mueller HintonII media (BD, NJ) containing 0.05% (v/v) Tween 80, 50 μg/ml Hyg, and 10 μg/ml Km with shaking at 115 r/min, 37 °C in a TAITEC BioShaker G・BR-300 (Tokyo, Japan). When the OD_600_ reached around 1.0, acetamide was added to a final concentration of 2%. Cells were harvested from 24 to 72 h after addition of acetamide by culture centrifugation at 15,000xg for 5 min at 4 °C.

### Determination of Mtb MDP1 (mMDP1) protein expression levels

An aliquot of cell pellet was first washed with distilled water. Glass beads (1 mm diameter, Kishida Chemical, Osaka, Japan) and Bugbuster HT Protein Extraction Reagent (Merck KGaA, Darmstadt, Germany) were then added to the cell pellet which was then disrupted with BeadSmash BS-12R (Wakenyaku, Kyoto, Japan) at 5500 rpm, 4 °C for 30 s three times with 30 s intervals between operation. Samples were then centrifuged at 14,000 rpm for 5 min at 4 °C. This was followed by separate collection of the supernatant and insoluble fractions, which were then treated with Laemmli sample buffer at 95 °C for 10 min. In some experiment, cells were disrupted with glass beads and water, and then treated with Laemmli sample buffer at 95 °C for 10 min. After centrifuging at 14,000 rpm for 5 min, the supernatant (whole cell lysate) was obtained. Sample’s protein concentrations were measured by Pierce™ BCA Protein Assay kit (Thermo Fisher Scientific, Waltham MA). The samples were electrophoresed on a sodium dodecyl sulphate (SDS)-polyacrylamide gel. Expression of mMDP1 was confirmed by SDS-PAGE and western blot analysis as described previously^12^.

### Extraction and purification of mMDP1

The cell pellet was disrupted by mortar and pestle with quartz sand (Kishida Chemical, Osaka, Japan). The disrupted sample was transferred to a glass beaker, and 0.25 M hydric acid was added and then stirred overnight at 4 °C. Cell suspension was centrifuged at 1,5000xg for 20 min at 4 °C, followed by transfer of the supernatant to a new breaker. The same volume of 8 M urea was added to supernatant, and then pH was adjusted to 8.0 with 10 M NaOH. For purification, two kinds of His binding buffer and two kinds of elution buffer were used.

The composition of His binding buffer used as 50 mM NaH_2_PO_4_, 500 mM NaCl, 10 mM Imidazole, 0.01% Tween 20, 6 M urea, pH = 8.0 [A1] and the same composition without urea [A2]. The composition of elution buffer was 50 mM NaH_2_PO_4_, 500 mM NaCl, 300 mM Imidazole, 0.01% Tween 20, 6 M urea [B1] and the same composition without urea [B2]. The supernatant was dialyzed against His binding buffer [A1] overnight. Dialyzed supernatant was filtered with 0.2 μm membrane and then 2-step purification was performed using fast performance liquid chromatography (FPLC, AKTA explorer 10 s, GE health care Japan, Tokyo, Japan). In the first step, the supernatant was absorbed onto a Ni column (HisTrap FF 5 ml, GE health care) with His binding buffer [A1], and then washed with 150 ml wash buffer {10% of [B1] and 90% of [A1]} at a flow rate of 2 ml/min. MDP1 adsorbed on the column was eluted with a gradient up to 100% [B1] at a flow rate of 2 ml/min. The presence of MDP1 in fractions were confirmed by SDS-Page (Supplementary Fig. S5A). In the second step, MDP1 was refolded on column. The fractions containing MDP1 were collected and then dialyzed against [A1]. MDP1-containing fractions were adsorbed on His Trap column and then refolded with 150 ml (30 column volumes) of His binding buffer [A2] at a flow rate of 1 ml/min. The refolded MDP1 were eluted with gradient up to 100% [B2] at a flow rate of 1 ml/min for 40 ml (8 column volumes), followed by elution with 25 ml of 100% [B2] (Supplementary Fig. 5B). Presence of MDP1 in the fractions was again confirmed by SDS-Page, and the MDP1-containing fractions were collected and dialyzed against saline. The obtained samples were confirmed by SDS-Page. LPS contamination in purified mMDP1 was below 0.1 EU/ml as assessed by Limulus test (Limulus Ambocyte Lysate QCL-1000, Lonza, Muenchensteinerstrasse, Switzerland).

### Extraction and purification of native MDP1 (nMDP1) from *Mycobacterium tuberculosis* H37RV

*M. tuberculosis* H37 was cultured in Sauton media^53^ for two weeks. Purification was performed in two steps similar to mMDP1, except for difference in composition of the buffer and column used (HiTrap Heparin HP, GE health care). Heparin buffer A (300 mM NaCl, 50 mM NaH_2_PO_4,_ 10 mM Imidazole, and 6 M Urea) and heparin buffer B (2 M NaCl, 50 mM NaH_2_PO_4,_ 10 mM Imidazole, and 6 M Urea) were utilized for nMDP1 purification, and heparin buffer A without urea and heparin buffer B without urea were used for on column refolding.

### Construction and purification of eMDP1

Since *mdp1 (hupB)* gene in *M. tuberculosis* contains rare codons for *E. coli*, we optimized the MDP1 sequence for expression in *E. coli* (Supllementary Fig. S3, Fasmac, Kanagawa, Japan)*.* The optimized or original sequence of MDP1 was inserted into pet22b vector with Nde1 site at N-terminus, and 6-His and Xho1 site at C-terminus, followed by transformation and amplification in DH5α. The plasmid was extracted by QIAprep Spin Miniprep Kit (Tokyo, Japan), and transformed into Rosetta2 (DE3) pLysS (Novagen, Germany), BL21Star (DE3) (Invitrogen, CA), or BL21 (DE3) pLysS (Invitrogen, CA), followed by plating on LB agar containing carbenicillin (50 μg/ml) and chloramphenicol (34 μg/ml). Obtained colony was cultured with shaking in LB broth containing antibiotics at 37 °C until OD_600_ 0.8, and then IPTG (f/c 0.5 mM) was added to induce the expression of MDP1. One and two hours later, 10 ml of the bacterial solution was taken, and then centrifuged at 8,000 rpm for 20 min at 4 °C. The bacterial cells were disrupted by using Bead Smash 12 (Wakenyaku, Kyoto, Japan) with glass beads (Tomy, Tokyo, Japan) and Bugbuster Protein Expression Reagent (Merck, Germany). The level of MDP1 expression was evaluated by SDS-PAGE and western blot by using 7C antibody (Supplementary Fig. S4). Purification of MDP1 was outsourced to YBIRD (Kihara memorial yokohama foundation, Kanagawa, Japan). Briefly, MDP1 expressed Rosetta2 (DE3) pLysS was cultured in the presence of IPTG for four hours. Bacterial cells were disrupted, and then, purified using HiTrap CM FF column (GE health care) and HiTrap Heparin HP column (GE health care). The LPS contamination in purified MDP1 was under 0.053 EU/ml assessed by Limulus test.

### Other *M. tuberculosis* antigens

Native Mtb MDP1 was isolated as previously described^11^. Some *M. tuberculosis* secreted antigens were purified from *M. tuberculosis* culture supernatant in Sauton media. The antigens were purified utilizing FPLC with DEAE-Sepholose column as descrived previously^31^. The obtained antigens were confirmed by SDS-PAGE and then analyzed by mass spectrometry (Triplet OF 5600^+^, SCIEX, Tokyo, Japan) after trypsin digestion and then verified by Mascot version 2.6 using the *M. tuberculosis* (Strain ATCC 25618/H37Rv) database^32^_._

### Chemical methylation of eMDP1

Chemical methylation of eMDP1 was performed according to the published method by Pethe  et al^17^ . Briefly, 800 µl of eMDP1 in 0.1 M borate buffer was treated with 35 µl of NaBH4 and 13 µl of 10 times diluted formalin on ice for 30 min. Methylated eMDP1 was confirmed by western blot with mAb38-8.

### CD spectra analysis and Sedimentation velocity analysis

We proceeded CD spectra analysis according to the same previously described method^13^. Briefly, recombinant eMDP1 and mMDP1were dialyzed in 50 mM sodium phosphate buffer (pH 7.0) containing 150 mM, 500 mM, or 1000 mM NaCl adjusted to a final concentration of 3.2 µM. The CD spectra was measured at 25 °C in cells that were 1 mm in width and recorded with a Jasco J-720. The proteins were dialyzed against phosphate buffer (pH 7.0) containing 150 mM, 500 mM, or 1000 mM NaCl prior to performing a run. The SV experiments were performed at 20 °C with an Optima XL-I (Beckman Coulter) using an An50Ti rotor. Concentration gradients were measured by UV absorption at 230 nm without a time interval. The partial specific volume of the protein, buffer density, and viscosity were calculated by Sednterp^13^. The distribution functions of the sedimentation coefficients, c(s), were calculated using the SEDFIT program, assuming that the frictional ratio was common to all molecular species. The c(s) was converted to the distribution of the molecular weights, c(M), based on the Svedberg equation, which was implemented in SEDFIT^13^.

### Glutaraldehyde crosslinking

We proceeded glutaraldehyde crosslinking according to a previously described method^13^. Briefly, recombinant proteins were separately incubated in 150 mM, 300 mM, 500 mM, 750 mM, 1000 mM, or 1500 mM NaCl solution at room temperature for 30 min and were crosslinked by the addition of glutaraldehyde to a final concentration of 0.2% or were left untreated. Samples were heated with Laemmli sample buffer at 95 °C for 5 min. The oligomerization of proteins was visualized by SDS-PAGE using 4–15% gradient acrylamide gel (Mini-PROTEAN TGX, Bio-Rad, CA).

### Study participants

Blood samples were obtained from 9 healthy students and staffs (one female and eight males) between the ages of 20 and 59 (average 29.7 ± 13.1) from 2017 to 2020 at Niigata University School of Medicine and the Graduate School of Medical and Dental Sciences. This study was approved by the Niigata University Ethics Committee (approval number 2015–2024) and was conducted according to its guidelines. The participants were recruited on campus and blood was drawn from those who consented. We confirmed BCG vaccine administration (BCG Tokyo 172 strain) during early childhood through a questionnaire, and individuals whom confirmation was not possible were excluded from this study.

### ODNs, G9.1, and Neg G9.1

G9.1 and Neg G9.1 were synthesized with a phosphodiester bond same as bacterial DNA (supplementary Table 1, Sigma Aldrich Japan, Tokyo, Japan). The phosphorothioate bonds in ODNs (Class A, B, C and their controls) were changed to phosphodiester bonds similar to G9.1 (Hokkaido system science, supplementary Table 2).

### Cellular responses to antigens

PBMC were isolated using Lymphoprep (STEMCELL techonologies, Vancouver, Canada) according to manufacturer’s protocol. 2 × 10^5^ cells were cultured with either 0.5 μM eMDP1, mMDP1, a combination of MDP1 and ODN (CpG-DNA) or other antigens. In some experiments, whole blood was cultured with addition of the same volume of RPMI containing 10% FCS and 2 mM L-glutamine, penicillin (100U/ml), streptomycin (100 μg/ml) and IL-2 (20 U/ml). Three or five days after cultivation, the supernatant was preserved at − 80 °C until IFN-gamma and IFN-alpha measurement. The measurement of IFN-gamma was performed by ELISA (BioLegend, San Diego, CA) according to manufacturer’s protocol.

### Biomolecular interaction measurements

The interaction between mMDP1 and peptides was monitored by measuring Surface Plasma Resonance (SPR) using a Biacore X100 (GE Healthcare Bio-Science AB, Uppsala, Sweden). All binding reaction were performed at 25 °C in HBS-EP (10 mM HEPES buffer, pH 7.4, containing 150 mM NaCl, 3 mM EDTA, and 0.05% Tween 20). mMDP1 was immobilized on the dextran matrix of the CM5 sensor chip (GE Healthcare Bio-Science AB, Uppsala, Sweeden) using the amine coupling kit (GE Healthcare Bio-Science AB, Uppsala, Sweeden) according to the manufacturer’s instructions. Association and dissociation rate constants were calculated using Biacore X100 Evaluation Software version 2.0.1. The interaction between mMDP1 and ODNs was performed by Bio-Layer Interferometry using Octet K2 (FORTEBIO, Sartorius, CA). MDP1 was immobilized on Nickel coated sensor chip (Ni–NTA), various concentrations of ODNs were solubilized in HBS-EP, and then plated in 96 well black flat plate (Greiner Bio One, Kremsmünstar, Austria). Data acquisition and Kinetics was performed using Data acquisition 8.1 and Data Analysis 8.1 software, respectively.

### Determination of post- translational modification of eMDP1 and mMDP1

The mass spectrometric analysis of MDP1 post-translational modifications was performed using limited proteolysis with trypsin, as indicated elsewhere^33^. Briefly, mMDP1 dissolved in 50 mM Tris HCl (pH 8.0) was digested with trypsin at a protein to trypsin ratio of 40 (weight/weight) at 37 °C for 2 min, 5 min, 10 min, and 16 h. The reaction was stopped by adding formic acid to a final concentration of 5% at the final concentration. Peptides were desalted by poly-stylendivinylbenzene (SDB) spin columns (GL Sciences, Tokyo, Japan), dried in a vacuum centrifuge, and stored at − 80 °C. The samples were analyzed by an Eksigent NanoLC415 nano-flow liquid chromatography system (Sciex, Framingham, MA, USA) coupled with a TripleTOF 5600 + tandem mass spectrometer (Sciex). The MS and MS/MS spectra were analyzed by Mascot version 2.6 (Matrix Science, Boston, MA, USA).

### Flowcytometry analysis

G9.1 was labeled with Cy5 (Hokkaido System Science, Sapporo, Japan). mMDP1 was labeled with Pacific Blue using Protein Labeling Kits according to manufacturer’s protocol (P30012, Thermo Fisher Scientific, Waltham, CA). Lineage cocktail (PE) was purchased from Beckman Coulter (Brea, CA), CD303 (FITC), CD123 (Brilliant Violet 650), HLA-DR (PE-Cy7), CD123 (Brilliant Violet 650) and CD11c were purchased from Biolegend (San Diego, CA). Dead cells were stained using Fixable Aqua Dead Cell Strain kit (L34957, Thermo Fisher Scientific, Waltham, CA). Isolated PBMCs (1 × 10^6^ ~ 2 × 10^6^/ml) were cultured with labeled mMDP1, G9.1 or a mixture of mMDP1 and G9.1 for 1 h at 5% CO2 and 37 °C. Cells were collected in tubes and treated with Human BD Fc Block (564219, BD Pharmingen, Franklin Lakes, NJ) to block Fc receptors, and then washed three timed with Staining medium (SM, Dulbecco’s PBS containing 2% FBS and 0.05% NaN_3_). Cell surface was stained with antibodies according to manufacturer’s protocol, followed by fixation with 4% paraformaldehyde in SM overnight. The following day, cells were analyzed by NovoCyte 3000 (ACEA Biociences, San Diego, CA).

### Statistics

Data was analyzed using Friedman tests followed by Dunn’s multiple comparisons test. All analysis was performed using GraphPad Prism 9.5.1 (GraphPad Software, Boston, MA, USA) and results with *p* < 0.05 were considered significant and marked by asterisks.

### Ethical approval

The experiments involving human blood collection were approved by the Niigata University Ethics Committee (approval number: 2015–2104) and were conducted in accordance with its guidelines. Informed consent was obtained from all participants. The experiments involving animals to obtain monoclonal antibodies were granted approval by the Niigata University Animal Experiment Committee (approved number: SA00073). The experiments were carried out in accordance with the committee’s guidelines and followed the ARRIVE guidelines.

### Supplementary Information

Supplementary Information.

## Data Availability

The original contributions presented in the study are included in the article/Supplementary materials. Further inquiries can be directed to the corresponding authors.
