# Supplementary material for: Recombinant mycobacterial DNA-binding protein 1 with post-translational modifications boosts IFN-gamma production from BCG-vaccinated individuals’ blood cells in combination with CpG-DNA
Source: Sci Rep. 2024 Apr 21;14:9141. doi: 10.1038/s41598-024-58836-8 (PMC11033290; doi:10.1038/s41598-024-58836-8)
Supplement: Supplementary file 1 — Supplementary Information. [file 41598_2024_58836_MOESM1_ESM.pdf]

Supplementary Information

Recombinant Mycobacterial DNA-binding Protein 1 with post-translational modifications boosts IFN-gamma production from BCG-vaccinated individuals' blood cells in combination with CpG-DNA

Yuriko Ozeki*, Akira Yokoyama, Akihito Nishiyama, Yutaka Yoshida, Yukiko Ohara, Tsukasa Mashima, Chikako Tomiyama, Amina K. Shaban, Atsuki Takeishi, Mayuko Osada-Oka, Takehiro Yamaguchi, Yoshitaka Tateishi, Jun-ichi Maeyama, Mariko Hakamata, Hiroshi Moro, Toshiaki Kikuchi, Daisuke Hayashi, Fumiko Suzuki, Toshiko Yamamoto, Sumiko Iho, Masato Katahira, Saburo Yamamoto, and Sohkichi Matsumoto*

Correspondence and requests for materials should be addressed to Y.O. (email:yuriozeki@med.niigata-u.ac.jp) and S.M. (email: sohkichi@med.niigata-u.ac.jp).

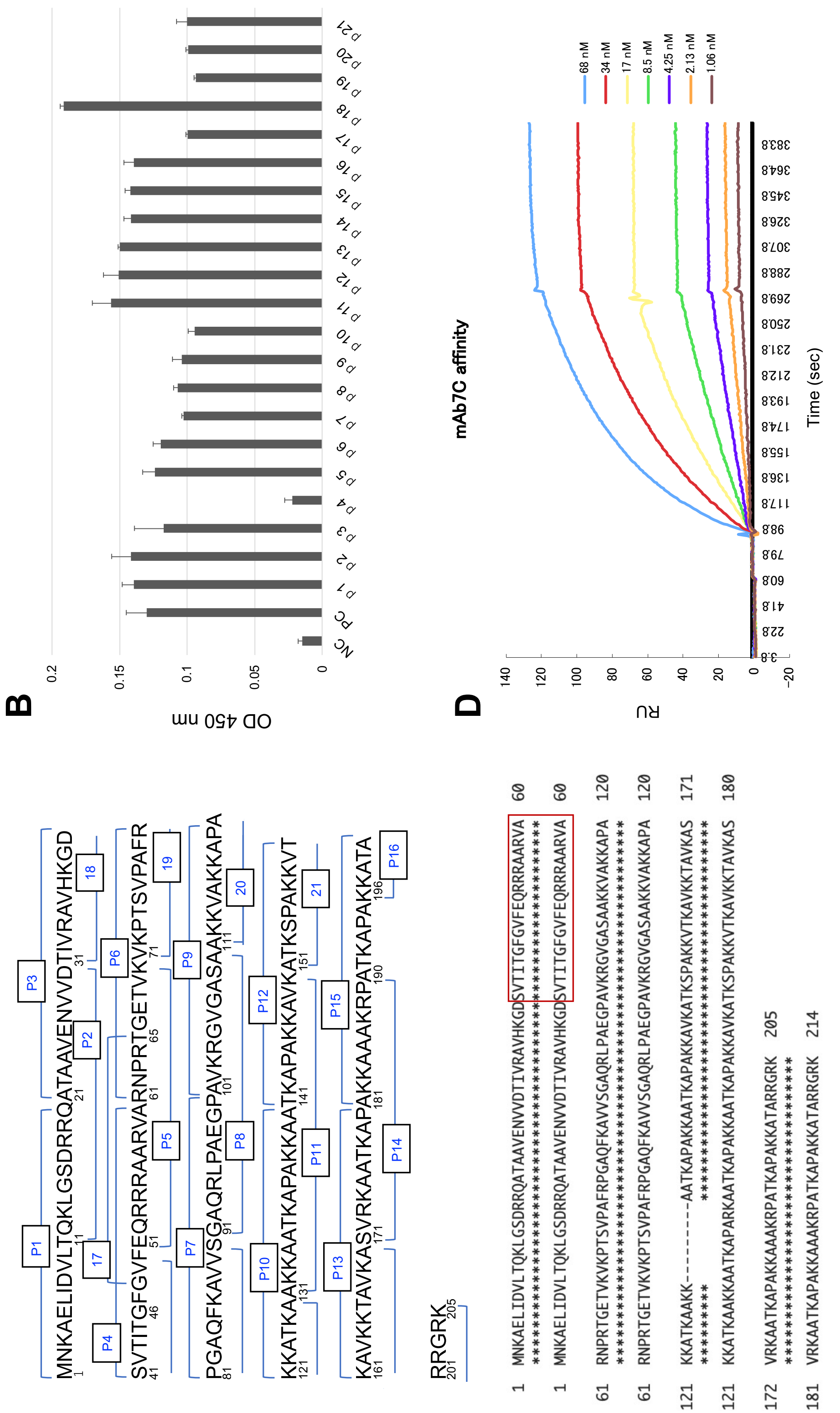

Supplementary Fig. S1. Ozeki et al.

Supplementary Fig. S1 mAb 7C recognizes amino acid 41-60 of MDP1.

(A) BCG MDP1 amino acid sequence. The sites of synthetic peptides and their corresponding code numbers are indicated (adapted from Furugen et al. Microb. Pathog. 2011).

(B) Determination of mAb binding sequences of MDP1. A mixture of each peptide and mAb 7C was plated on an MDP1 coated plate. A horseradish peroxidase (HRP) conjugated secondary antibody was added, and peroxidase activity was determined by measuring absorbance at OD450 nm.

(C) MDP1 amino acid (AA) sequence. Upper: BCG MDP1 AA sequence. Lower: Mtb MDP1 AA sequence. The sequence enclosed by a square is recognized by mAb 7C.

(D) SPR analysis of the interaction between mAb 7C and MDP1 purified from BCG. 140 ng of MDP1 was immobilized on a CM5 sensor chip, and sequential molar amounts of mAb 7C ranging from1.06 to 68 nM were loaded for 200 sec. The SPR analysis was conducted for a total of 420 sec. The calculated KD value was 3.08e-10 M.

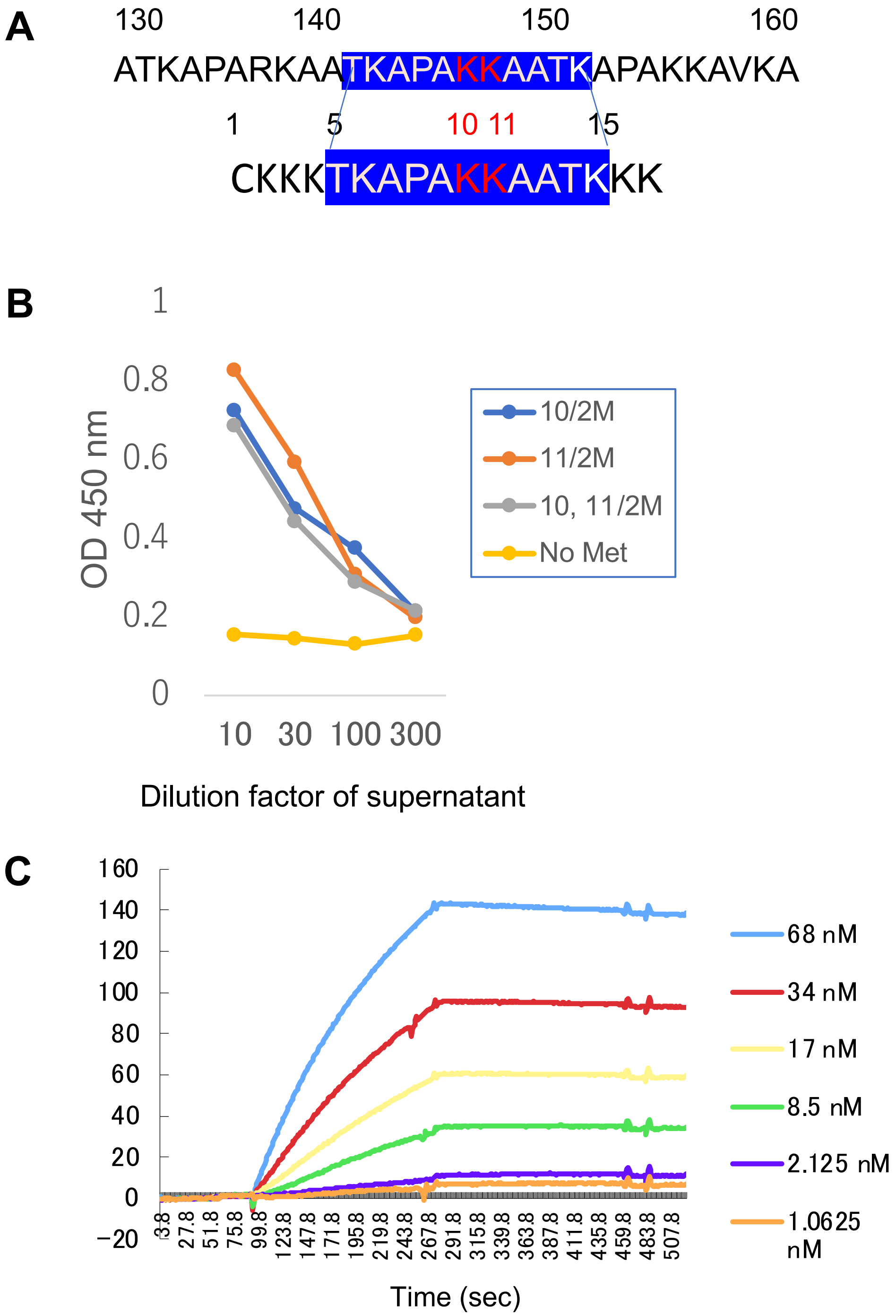

Supplementary Fig. S2　Ozeki et.al

Supplementary Fig. S2 mAb 38-8 specifically recognizes methylated MDP1.

(A) Peptide sequence used for antibody production. The peptide sequence used to immunize mice (lower) and its corresponding MDP1 portion (upper).

(B) ELISA results of supernatant from 38-8 hybridoma. Diluted supernatant from the 38-8 hybridoma was incubated with BSA-conjugated peptides. All synthetic peptides had a similar sequence: CKKKTKAPA(10)K(11)KAATKKK. However, 10/2M, 11/2M, and 10, 11/2M peptides are dimethylated at 10K, 11K, and both 10 and 11 K resides, respectively. No Met refers to a peptide without methylation. The vertical axis represents IgG levels measured at OD450 nm.

(C) SPR analysis of the interaction between mAb 38-8 and MDP1 purified from *M. tuberculosis*. 140 ng of MDP1 was immobilized on a CM5 sensor chip, and sequential molar amounts of mAb 38-8 ranging from 1.06 to 68 nM were loaded for 200 sec. The SPR analysis was conducted for a total of 420 sec. The calculated KD value was 7.56e-9 M.

A

```
Original      1  CATATGAACAAAGCAGAGCTCATTGACGTGCTCACACAGAAATTGGGCTCGGACCGTCGG  60
                 ****************** * ********  * ** ***** *****    ** ** **
Optimized     1  CATATGAACAAAGCAGAGTTGATTGACGTCTTAACCCAGAAGTTGGGGAGCGATCGCCGC  60

             61  CAGGCGACCGCCGCCGTCGAGAATGTCGTTGACACGATTGTGCGTGCGGTACACAAAGGC  120
                 *********** ******** ***** ***** ** ** ** ** ** ** ** *****
             61  CAGGCGACCGCAGCCGTCGAAAATGTGGTTGATACCATCGTCCGCGCCGTTCATAAAGGT  120

            121  GACAGCGTCACCATTACCGGGTTCGGTGTGTTCGAACAGCGTCGCCGCGCGGCTCGAGTG  180
                 **    ** ** ***** ** ** ** ** ** ***************** ** ** **
            121  GATTCTGTAACGATTACGGGCTTTGGGGTCTTTGAACAGCGTCGCCGCGCAGCGCGTGTA  180

            181  GCCCGCAATCCGCGTACCGGCGAGACAGTAAAGGTGAAGCCGACGTCGGTGCCGGCGTTC  240
                 ** ***** ** ***** ** ** ***** ***** ***** ** ***** ***** **
            181  GCGCGCAACCCACGTACTGGTGAAACAGTTAAGGTTAAGCCAACCTCGGTTCCGGCCTTT  240

            241  CGCCCGGGCGCGCAATTCAAAGCGGTTGTGTCTGGCGCGCAGCGTCTCCCGGCAGAAGGA  300
                 ***** ***** *********** ******  ****** ** ** ** ***** ** **
            241  CGCCCAGGCGCACAATTCAAAGCCGTTGTGAGTGGCGCTCAACGCCTGCCGGCGGAGGGT  300

            301  CCCGCTGTTAAGCGTGGTGTGGGGGCCAGTGCAGCCAAGAAGGTAGCGAAGAAGGCACCT  360
                 ** ** ** ***** ** ***** ***   ** ** ******** *********** **
            301  CCTGCGGTGAAGCGCGGCGTGGGTGCCTCAGCTGCAAAGAAGGTGGCGAAGAAGGCGCCA  360

            361  GCCAAGAAGGCGACAAAGGCCGCCAAGAAGGCGGCGACCAAGGCGCCCGCCAGGAAGGCG  420
                 ** *********** ***** ** ******** ** ** ** ** ** **  * ******
            361  GCGAAGAAGGCGACGAAGGCAGCAAAGAAGGCAGCCACTAAAGCTCCGGCTCGCAAGGCG  420

            421  GCGACCAAGGCGCCCGCCAAGAAAGCGGCGACCAAGGCGCCCGCCAAGAAAGCTGTCAAG  480
                 ** ** ** ** ** ** ******** ** ***** ** ** *********** ** **
            421  GCAACAAAAGCACCGGCGAAGAAAGCCGCTACCAAAGCTCCGGCCAAGAAAGCGGTGAAA  480

            481  GCCACGAAGTCACCCGCCAAGAAGGTGACCAAGGCGGTGAAGAAGACCGCGGTCAAGGCA  540
                 ** ** **    ** ******** ** ** ** ** ******** ** ** ** ** **
            481  GCGACCAAAAGTCCAGCCAAGAAAGTAACTAAAGCTGTGAAGAAAACGGCAGTGAAAGCG  540

            541  TCGGTGCGTAAGGCGGCGACCAAGGCGCCGGCAAAGAAGGCAGCGGCCAAGCGGCCGGCT  600
                 ** ** ** ** ** ** ** ** ******** ***** ** ******** ** ** **
            541  TCCGTCCGCAAAGCCGCAACTAAAGCGCCGGCCAAGAAAGCTGCGGCCAAACGCCCAGCG  600

            601  ACCAAGGCTCCCGCCAAGAAGGCAACCGCTCGGCGGGGTCGCAAACTCGAG  651
                 ** ** ** ** ** ***** ** ** ** ** ** ** ** *********
            601  ACGAAAGCGCCTGCAAAGAAAGCCACGGCACGCCGTGGCCGTAAACTCGAG  651
```

B

```
Original      1  MNKAELIDVLTQKLGSDRRQATAAVENVVDTIVRAVHKGDSVTITGFGVFEQRRRAARVA  60
                 ************************************************************
Optimized     1  MNKAELIDVLTQKLGSDRRQATAAVENVVDTIVRAVHKGDSVTITGFGVFEQRRRAARVA  60

             61  RNPRTGETVKVKPTSVPAFRPGAQFKAVVSGAQRLPAEGPAVKRGVGASAAKKVAKKAPA  120
                 ************************************************************
             61  RNPRTGETVKVKPTSVPAFRPGAQFKAVVSGAQRLPAEGPAVKRGVGASAAKKVAKKAPA  120

            121  KKATKAAKKAATKAPARKAATKAPAKKAATKAPAKKAVKATKSPAKKVTKAVKKTAVKAS  180
                 ************************************************************
            121  KKATKAAKKAATKAPARKAATKAPAKKAATKAPAKKAVKATKSPAKKVTKAVKKTAVKAS  180

            181  VRKAATKAPAKKAAAKRPATKAPAKKATARRGRK  214
                 **********************************
            181  VRKAATKAPAKKAAAKRPATKAPAKKATARRGRK  214
```

Supplementary Fig. S3 Ozeki et al.

Supplementary Fig. S3 Optimized and original sequences of MDP1 (hupB).

(A) Original and optimized DNA sequences of *M. tuberculosis* MDP1 (hupB) with Nde1 site (CATATG) at the N terminal, and Xho1 site (CTCGAG) at the C terminal. upper: original sequence, lower: optimized sequence. square: restriction site. ＊：indicate base matching.

(B) Translated amino acid sequences. upper: translated amino acid sequence of the original MDP1 DNA. lower: translated amino acid sequence of optimized DNA. *: indicate amino acid matching.

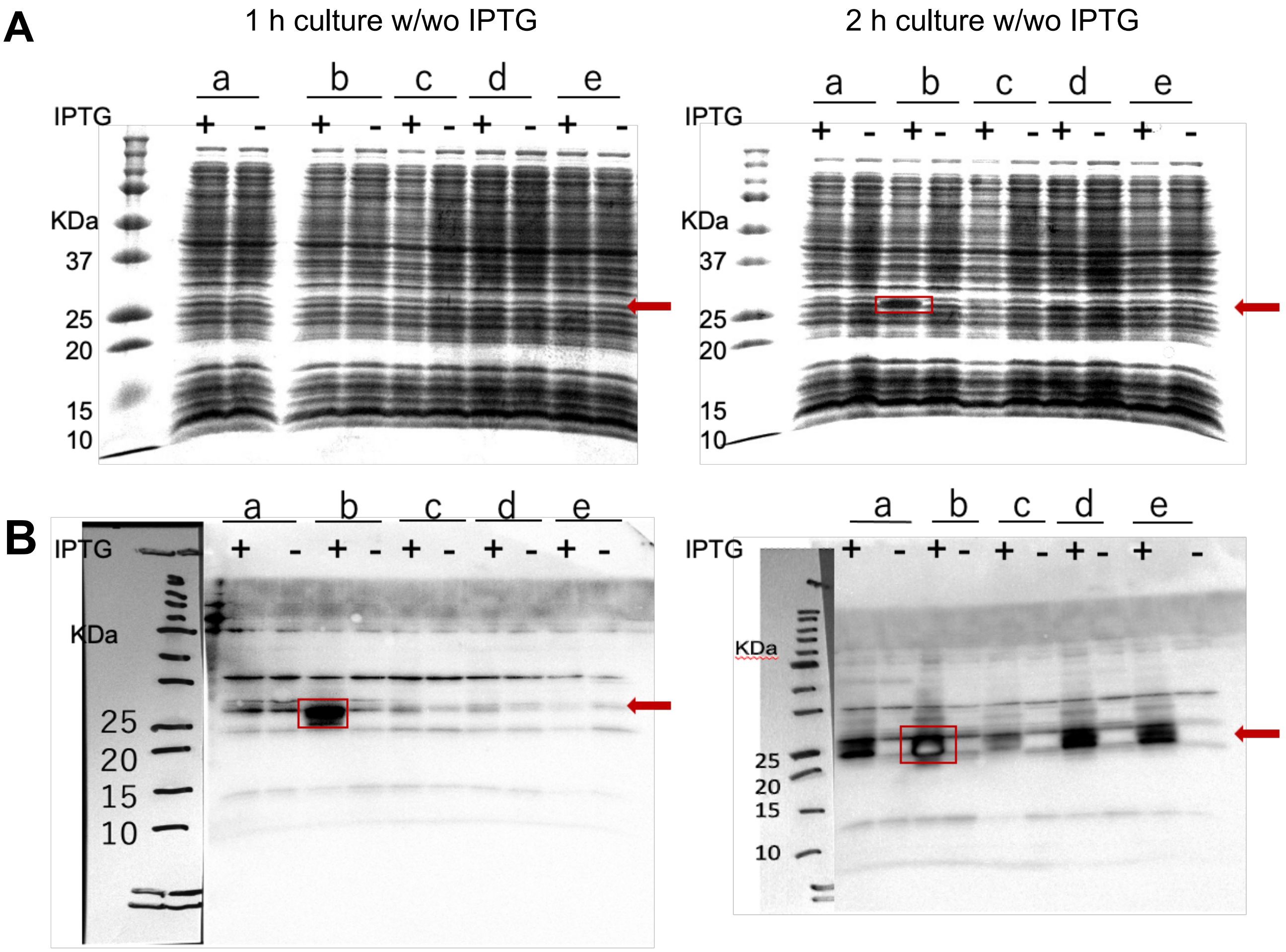

Supplementary Fig. S4 Ozeki et al.

Supplementary Fig. S4 Differences in MDP1 expression levels in *E. coli* host.

pET22b vector containing either the optimized or original MDP1 sequence was transformed into Rosetta2 (DE3) pLysS, BL21Star (DE3), or BL21 (DE3) pLysS *E. coli* strains. Colonies were obtained and cultured by shaking in LB broth containing carbenicillin (50 µg/ml) and chloramphenicol (34 µg/ml) at 37 °C until OD_{600} 0.8. IPTG (f/c 0.5 mM) was then added to induce the expression of MDP1. After one and two hours, 10 ml of bacterial solution was harvested and centrifuged at 8,000 rpm for 20 min at 4°C. Bacterial cells were disrupted using Bead Smash 12 (ku, Kyoto, Japan) with glass beads (Tomy, Tokyo, Japan) and Bugbuster Protein Expression Reagent (Merck, Germany). 30 µg of bacterial protein was analyzed by SDS-PAGE (A) and western blot (B) with anti-His antibody.

A, CBB stain. B, Western blot with anti-His antibody. a: MDP1/pet22b/BL21(DE3) pLysS, b: MDP1/pet22b/Rosetta2 (DE3) pLysS, c: MDP1/pet22b/BL21Star (DE3), d: MDP1(optimised)/pet22b/ BL21(DE3) pLysS, e: MDP1(optimised)/pet22b/BL21Star (DE3). +: IPTG added, -: control. Arrows indicated MDP1 expression bands.

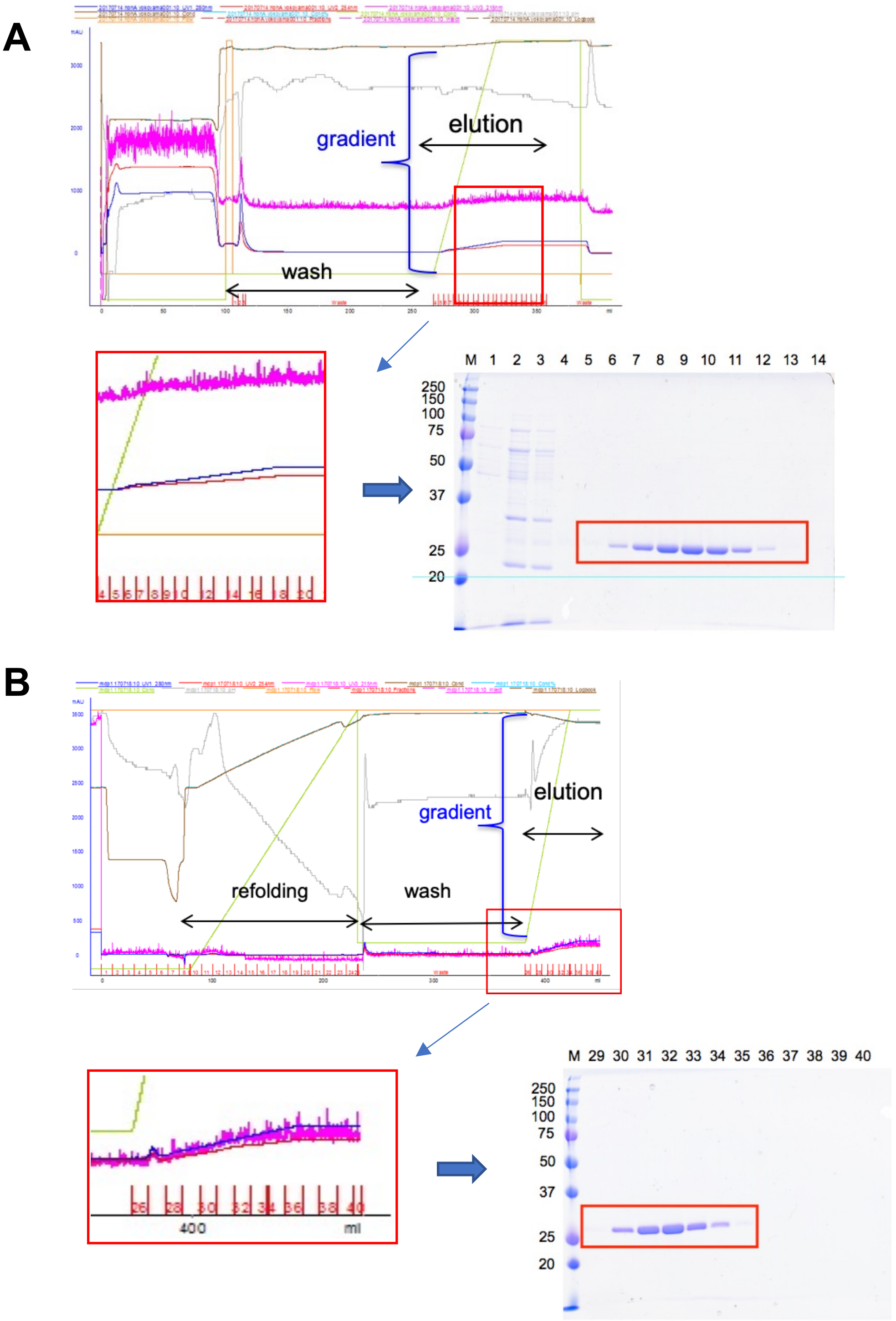

Supplementary Fig. S5 Ozeki et al.

Supplementary fig. S5 Two-step Purification of mMDP1 by FPLC.

(A) Bacterial culture was harvested 48 h after ACE addition.

Protein was disrupted with quartz sand and then extracted with 0.25 N HCl. After neutralization with NaOH, the fractions containing MDP1 were dialyzed to His binding buffer containing 6 M urea, followed by purification using FPLC. Proteins were applied to a nickel column in His binding buffer containing 6 M urea, and then unbound proteins were washed with a mixture of 90% His binding buffer and 10% elution buffer. Column-bound proteins containing MDP1 were eluted with elution buffer with 6 M urea (upper panel). The obtained fractions were analyzed by SDS-PAGE (lower panels).

(B) Refolding step. Fractions containing MDP1 were dialyzed against His binding buffer containing 6 M urea, and then applied to a nickel column. MDP1 was refolded using a linear gradient of urea from 6 M to 0 on column, and then eluted with elution buffer (upper panel). Fractions were analyzed by SDS-PAGE (lower panels).

Supplementary table 1. Types and sequences of ODNs.

Name of Oligo	Type	Sequence
G9.1	new class	5'-GGGGGGGGGGACGA:TCGTCG-3'
Neg G9.1	new class	5'-GGGGGGGGGGAGCA:TGCTCG-3'
ODN 2216	Class A	5’-GGGGGACGA:TCGTCGGGGGG-3’
ODN 2216 control	Class A	5’-GGGGGAGCA:TGCTGGGGGGG-3’
ODN 2006	Class B	5’-TCGTCGTTTTGTCGTTTTGTCGTT-3’
ODN 2006 control	Class B	5’-TGCTGCTTTTGTGCTTTTGTGCTT-3’
ODN 2935	Class C	5’-TCGTCGTTTTCGGCGC:GCGCCG-3’
ODN 2935 control	Class C	5'-TGCTGCTTTTGGGGGG:CCCCCC-3'

underline, palindrome

Supplementary table 2. Affinity of mMDP1 with ODNs.

Immobilized ligand	Analytes	Association rate, ka	Dissociation rate, kd	Affinity, KD
		1/Ms	1/s	M
mMDP1	G9.1	2.96×10^{-4}	1.99×10^{-4}	6.73×10^{-9}
	Neg G9.1	4.29×10^{-4}	2.11×10^{-4}	4.93×10^{-9}
	Class A	1.47×10^{-4}	3.67×10^{-4}	2.50×10^{-8}
	control	2.36×10^{-4}	2.56×10^{-4}	1.08×10^{-8}
	Class B	1.71×10^{-4}	9.95×10^{-4}	5.83×10^{-8}
	control	Nt	Nt	Nt
	Class C	-	-	-
	control	Nt	Nt	Nt

-, not bound; Nt, not tested

Supplementary Fig.S6
(Full length blot of Fig.1 B and Fig.1 D are indicated)

Fig.1 B middle right

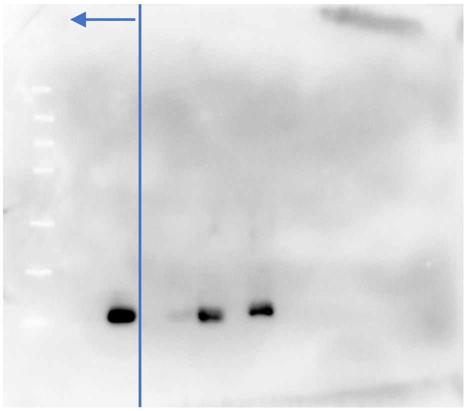

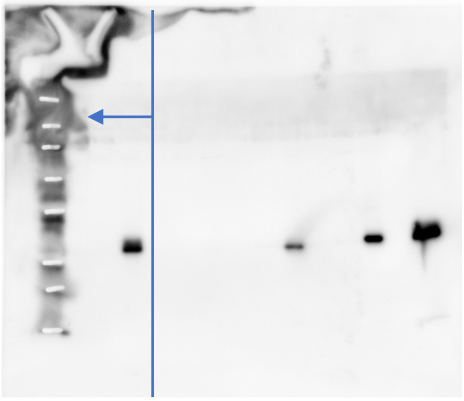

Fig.1 D left

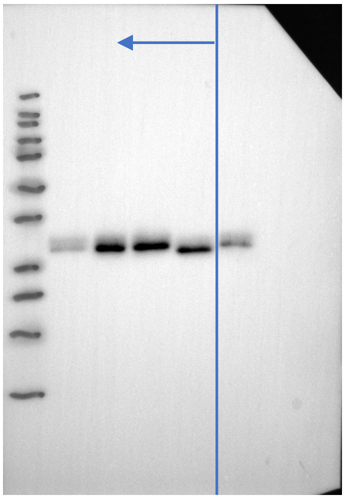

The left portion of the blue lines was included in Fig.1.
